# Effects of rutin on renal function, oxidative stress and fibrosis in animal models of diabetic nephropathy: a systematic review and meta-analysis

**DOI:** 10.3389/fphar.2026.1771010

**Published:** 2026-02-23

**Authors:** Zongtao Li, Yashi Wang, Die Fang, Yiman Wang, Hongzhe Han, Xueqin Zhang, Zhiqiang Chen

**Affiliations:** Hebei University of Chinese Medicine, Shijiazhuang, China

**Keywords:** animal model, diabetic nephropathy, meta-analysis, oxidative stress, preclinical study, renal fibrosis, rutin

## Abstract

**Background:**

Diabetic nephropathy is a major microvascular complication of diabetes and a leading cause of end-stage renal disease, while effective disease-modifying therapies remain limited. Rutin, a naturally occurring flavonoid with antioxidant and anti-inflammatory properties, has shown renoprotective effects in experimental diabetic nephropathy; however, its overall efficacy has not been quantitatively synthesized. This study aimed to systematically evaluate the preclinical effects of rutin in animal models of diabetic nephropathy.

**Methods:**

A systematic review and meta-analysis of preclinical animal studies was conducted following PRISMA guidelines and prospectively registered in INPLASY (INPLASY2025110085). Six English and Chinese databases, including PubMed and Web of Science, were searched from inception to November 2025. Studies assessing rutin monotherapy in diabetic nephropathy models were included. Primary outcomes were serum creatinine, blood urea nitrogen, and 24-h urinary protein. Secondary outcomes included blood glucose, lipid parameters, oxidative stress markers, and fibrosis-related indicators. Risk of bias was assessed using SYRCLE’s tool, and pooled effect sizes were calculated using standardized mean differences with Stata 17.0.

**Results:**

Thirteen studies involving 318 animals met the inclusion criteria. Meta-analysis showed that rutin significantly reduced serum creatinine, blood urea nitrogen, and 24-h urinary protein levels, indicating improved renal function. Rutin also lowered blood glucose and lipid levels. In addition, rutin attenuated oxidative stress by reducing reactive oxygen species and malondialdehyde while enhancing endogenous antioxidant defenses. Profibrotic markers, including transforming growth factor-β, were also significantly decreased. Sensitivity analyses demonstrated that the pooled estimates were not driven by any single study, while subgroup analyses suggested that differences in study characteristics may partially contribute to heterogeneity without altering the overall direction of effects.

**Conclusion:**

This meta-analysis provides quantitative preclinical evidence that rutin exerts broad renoprotective effects in experimental diabetic nephropathy through coordinated regulation of metabolic disturbance, oxidative stress, and fibrosis. These findings support rutin as a potential multi-target candidate for further mechanistic investigation and translational research.

**Systematic Review Registration:**

https://inplasy.com/inplasy-2025-11-0085, identifier INPLASY2025110085.

## 1 Introduction

Diabetic nephropathy (DN) is a major microvascular complication of diabetes and a leading cause of end-stage renal disease (ESRD) (Zhang et al., 2025). Clinically, DN is characterized by persistent proteinuria, hypertension, and progressive renal dysfunction. According to the 10th edition of the IDF Diabetes Atlas, the global diabetic population reached approximately 540 million in 2021 and is projected to increase to 780 million by 2045, underscoring the growing public health burden of DN (Sun et al., 2022). Despite current therapeutic strategies, including glycemic control and renin–angiotensin–aldosterone system blockade, many patients continue to experience progressive renal decline, highlighting the urgent need for more effective treatment options (Rossing et al., 2022a; Rossing et al., 2022b).

**FIGURE 1:**
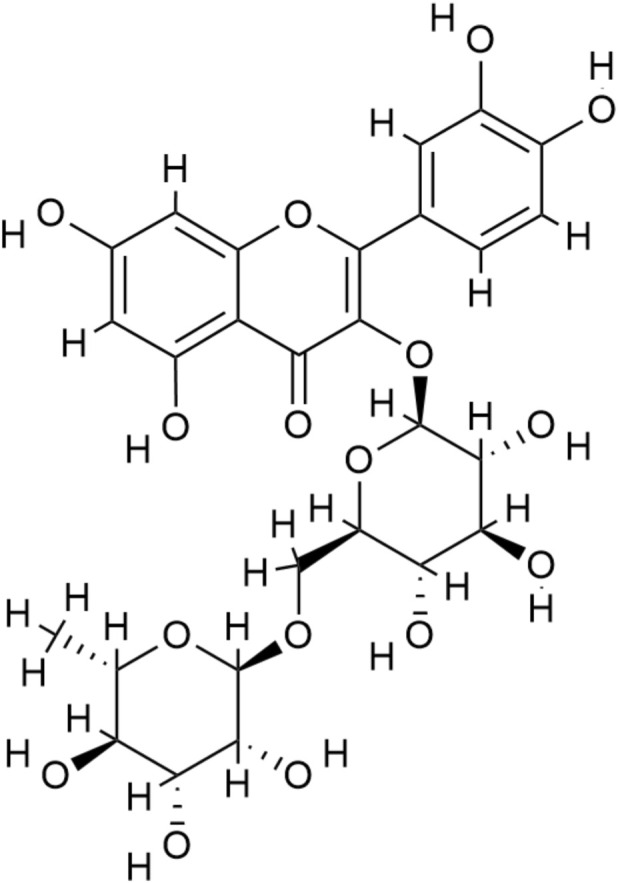
The chemical structure of rutin.

Rutin (also referred to as quercetin-3-rutinoside) is a natural flavonoid compound that is present in large quantities in buckwheat, citrus fruits, and other plants, which are widely used as sources of food and medicine. Its molecular formula is C_27_H_30_O_16_ (Figure 1), and its structure consists of a quercetin core linked to a rutinose sugar moiety. Previous pharmacological studies have shown that rutin has various biological effects, such as those of antioxidant (Tabolacci et al., 2023), anti-inflammatory (Jang et al., 2025), improvement of lipid-metabolism (David et al., 2023), stabilization of endothelial (Serri et al., 2024), and control of platelet functions (Choi et al., 2021; Karunarathne et al., 2023). These properties render rutin a promising candidate for the treatment of metabolic kidney injury, particularly DN. Notably, rutin is structurally related to quercetin, but it cannot be considered a replacement for quercetin in DN. The rutinose molecule can have a significant effect on solubility and intestinal processing, and result in various systemic and renal exposure characteristics. Rutin may act *in vivo* in both the parent glycoside and in metabolites (such as quercetin) produced in the process of digestion and metabolism, which explains why it is important to assess rutin as an intervention in itself and not to conclude about its efficacy on the basis of quercetin or other flavonoids. Accordingly, recent years have seen a gradual increase in animal studies investigating rutin in DN models. Existing evidence regarding the effectiveness of rutin as an agent for treating DN, however, is largely based on small-sample animal studies, undertaken in various labs, with extensive differences in models, doses, periods of intervention and end point measures. As a result, the overall efficacy and mechanisms of rutin in DN have not yet been systematically evaluated.

Systematic reviews and meta-analyses are essential evidence-based research methods. They integrate existing evidence under a unified framework, which enhances statistical power, reduces bias from individual studies, and provides more reliable data for potential clinical applications (Page et al., 2021). Compared to single animal studies, a systematic review can offer a more comprehensive view of rutin’s renal protective effects in DN. It also allows for a deeper exploration of dose dependence, intervention duration, and potential differences in mechanisms. Thus, to evaluate the effect of rutin as a renal protective agent in DN animal models and to summarize its possible pharmacological mechanisms, a systematic review and meta-analysis of relevant literature should be designed properly, with transparency and data-driven background.

## 2 Methods

### 2.1 Systematic review registration

To guarantee the rigor and traceability of the study design, this study was performed according to the PRISMA guidelines of systematic reviews and meta-analyses (JVDS et al., 2023), with adaptations appropriate for preclinical animal studies. It was registered in INPLASY before the start of the study (registration number: INPLASY2025110085).

### 2.2 Data sources and search strategy

**FIGURE 2:**
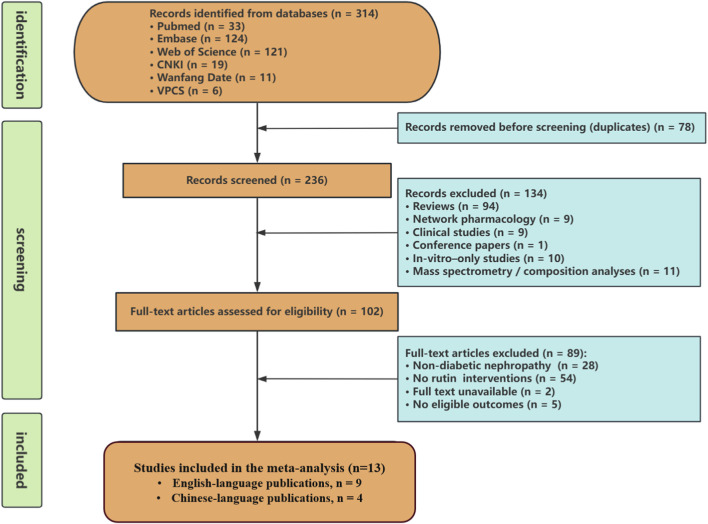
Selection of studies for the meta-analysis.

To ensure the comprehensiveness and reliability of the meta-analysis results, this study systematically searched both English and Chinese databases to gather all relevant preclinical evidence. The English sources included PubMed, Web of Science, and Embase, while the Chinese sources covered CNKI, Wanfang, and VIP databases. The search was conducted for all publications available up to November 2025. The search framework integrated both thesaurus-based terms and unrestricted text words. Diabetic kidney disease–related terminology—namely “Nephropathies, Diabetic”, “Nephropathy, Diabetic”, “Diabetic Kidney Disease”, “Diabetic Kidney Diseases”, “Kidney Disease, Diabetic”, “Kidney Diseases, Diabetic”, “Diabetic Nephropathy”, “Diabetic Glomerulosclerosis”, “Glomerulosclerosis, Diabetic”, “Intracapillary Glomerulosclerosis”, “Kimmelstiel-Wilson Disease”, “Kimmelstiel Wilson Disease”, “Nodular Glomerulosclerosis”, “Glomerulosclerosis, Nodular”, “Kimmelstiel-Wilson Syndrome”, “Kimmelstiel Wilson Syndrome”, and “Syndrome, Kimmelstiel-Wilson”—was matched with compound-specific identifiers, including “Rutin”, “Rutoside”, “Quercetin-3-Rutinoside”, “Quercetin 3 Rutinoside”, “3-Rhamnosyl-Glucosyl Quercetin”, and “Quercetin, 3-Rhamnosyl-Glucosyl”. Full search details are available in Figure 2 and the Supplementary Material.

### 2.3 Inclusion and exclusion criteria

This study followed the PICO framework to establish inclusion criteria. Studies meeting the following conditions were considered for inclusion: (Zhang et al., 2025): animal models of DN; (Sun et al., 2022); interventions using rutin monomer, with at least one dosage group; (Rossing et al., 2022a); a DN model control group, receiving either a placebo or no treatment; (Rossing et al., 2022b); reporting key renal outcome measures, including serum creatinine (Scr), blood urea nitrogen (BUN), and 24-h urinary protein (24-h UTP). If studies also provided data on blood glucose, lipid levels, oxidative stress, or fibrosis biomarkers, these were extracted as secondary outcomes. Exclusion criteria included: (Zhang et al., 2025): non-animal studies, such as clinical trials, reviews, case reports, or *in vitro* studies; (Sun et al., 2022); studies that met inclusion criteria but were not accessible in full text; (Rossing et al., 2022a); studies lacking an independent control group, or those with combined treatments making it impossible to isolate the effects of rutin; (Rossing et al., 2022b); duplicate publications; (Tabolacci et al., 2023); studies without the necessary data for quantitative analysis, such as sample size, standard deviation, or standard error.

### 2.4 Study selection and data extraction

Literature search, study selection, data extraction and quality assessment were conducted by two reviewers (Zongtao Li and Yashi Wang) based on the predetermined inclusion and exclusion criteria. Any discrepancies were resolved through discussion or by consulting the corresponding author.

The step of data extraction involved the reviewers separately gathering the following key pieces of information: (Zhang et al., 2025): publication date and first author, (Sun et al., 2022), animal characteristics, such as species, sex, age, and weight, (Rossing et al., 2022a), methods of establishing the diabetes model, including induction drugs, route of administration, doses, modeling duration, and diagnostic criteria, (Rossing et al., 2022b), intervention factors in both experimental and DN model groups, such as administration method, dose, and duration, and (Tabolacci et al., 2023) primary and secondary outcome measures. For studies with co-interventions or multi-component designs, only comparisons between the rutin monotherapy arm and the DN model control arm (without additional active treatment) were extracted to isolate the effect of rutin; combined-treatment arms were not synthesized. When multiple control arms were available, the control arm with an identical background regimen (except rutin) was preferentially selected. For multi-arm animal studies with multiple rutin doses but a single shared control group, we followed the guidance on multi-arm trials from the Cochrane Handbook (sixth edition) (Chndler, 2024). The sample size of the shared control group was split as evenly as possible among the comparisons, ensuring the total sample size matched the original control group size to avoid double-counting the same control animals, which would artificially inflate the study’s weight. During this process, the mean and standard deviation of the control group remained unchanged, and only the sample size was adjusted to ensure reasonable weight distribution. In studies that reported data at multiple time points, the most recent measurement obtained before euthanasia was used.

In cases where study results were provided only in the form of graphs, we first attempted to contact the authors to obtain the original data. If no response was received, we used WebPlotDigitizer 4.8 software to extract data from the graphs. To ensure the accuracy of the data, the reviewers digitized the data separately and cross-checked the results. Any discrepancies were resolved through consultation or discussion with the respective author. Results presented as standard error of the mean (SEM) were converted to standard deviation (SD) using the formula SD = SEM × √n.

### 2.5 Risk of bias

The risk of bias in the included studies was independently assessed using the SYRCLE risk of bias assessment tool for animal intervention studies (Carlijn et al., 2014). This tool is specifically designed for methodological evaluation of animal studies and consists of 10 items aimed at identifying potential sources of systematic bias during the research process. The assessment covers the following areas: (Zhang et al., 2025): random sequence generation; (Sun et al., 2022); comparability of baseline characteristics; (Rossing et al., 2022a); allocation concealment; (Rossing et al., 2022b); random housing; (Tabolacci et al., 2023); blinding during the study (performance bias); (Jang et al., 2025); random outcome assessment; (David et al., 2023); blinding of outcome measurements (detection bias); (Serri et al., 2024); completeness of outcome data; (Choi et al., 2021); selective reporting; and (Karunarathne et al., 2023) other potential sources of bias. Disagreements during the assessment were resolved through discussion or consultation with the corresponding author. Additionally, a stratified analysis was performed by categorizing studies based on their risk-of-bias scores from the SYRCLE tool. Pooled effect sizes were calculated separately for each risk-of-bias group to explore whether studies with higher risk-of-bias scores reported larger effects compared to those with lower scores.

### 2.6 Subgroup analysis

To investigate the potential impact of different study characteristics on effect sizes, this study pre-defined four subgroups: (Zhang et al., 2025): animal species; (Sun et al., 2022); modeling methods for DN; (Rossing et al., 2022a); duration of rutin intervention; and (Rossing et al., 2022b) rutin dosage. Subgroup analyses were primarily exploratory and aimed at identifying possible sources of heterogeneity when significant variation was observed. All subgroup analyses were performed using the Meta-Analysis module in Stata 17 via the graphical interface.

### 2.7 Statistical analysis

All statistical analyses were conducted using Stata 17.0. The data included in this study were continuous outcomes, and the overall effect size was represented by the standardized mean difference (SMD), with 95% confidence intervals (CIs) used to indicate the uncertainty of the estimates. Heterogeneity was evaluated using the I^2^ statistic. An I^2^ value greater than 50% indicates significant heterogeneity, and in this case, a random-effects model (DerSimonian–Laird method) was used. When I^2^ was low, a fixed-effects model was applied. All analyses were performed through the Meta-Analysis menu in Stata 17, without the need for command inputs. Statistical significance was considered at *P* < 0.05. Given the generally unclear or high risk of selection bias, including the lack of reported allocation concealment in the included studies, pooled effect sizes were interpreted with caution. The purpose of quantitative synthesis was to summarize the overall direction and consistency of effects across studies rather than to provide precise or unbiased estimates of treatment efficacy.

### 2.8 Publication bias

To assess the risk of publication bias for the primary outcomes, a funnel plot was created, and Egger’s regression test was performed. These analyses were conducted using the Publication Bias function in the Meta-Analysis module of Stata 17. When Egger’s test suggested potential bias, the trim-and-fill method was further applied to estimate the missing studies and obtain an adjusted pooled effect.

### 2.9 Sensitivity analysis

To assess the robustness of the meta-analysis results, sensitivity analysis was performed using the “leave-one-out analysis” function in the Meta-Analysis module of Stata 17. This method involves systematically excluding each study and recalculating the pooled effect to determine if any individual study significantly impacts the overall effect size. If the pooled effect remains largely unchanged after each exclusion, the results are considered to be robust.

### 2.10 Dose-time-response analysis

To explore the effects of varying dosing schedules and treatment durations on key changes in DN, this paper created three-dimensional dose-time response plots for Scr, BUN, and 24-h UTP based on available literature. This visualization analysis was used to illustrate the relationship between rutin dosage, treatment duration, and changes in efficacy, providing a clearer method to determine the potential differences in the effects of different dosing strategies on improving kidney function.

## 3 Results

### 3.1 Study selection

A total of 314 articles were retrieved from six databases in this study, including PubMed (33 articles), Embase (124 articles), Web of Science (121 articles), CNKI (19 articles), Wanfang (11 articles), and VIP (6 articles). After merging the search results and removing 78 duplicate records, 236 articles were included in the initial screening. Based on title and abstract screening, 134 articles were excluded for not meeting the inclusion criteria. The main reasons for exclusion were: review articles (94), network pharmacology studies (Choi et al., 2021), clinical studies (Choi et al., 2021), conference abstracts (Zhang et al., 2025), *in vitro* studies (Karunarathne et al., 2023), and mass spectrometry/compound analysis studies (Page et al., 2021). Subsequently, 102 articles underwent full-text assessment, and 89 articles were further excluded. The reasons for exclusion included: non-DN models (Tanase et al., 2022), studies not using rutin as an intervention (Wu et al., 2021), full text unavailable (Sun et al., 2022), and studies not reporting the predefined primary outcomes (Tabolacci et al., 2023). Ultimately, 13 studies met the inclusion criteria and were included in the meta-analysis (see Figure 2), comprising 9 English-language and 4 Chinese-language publications. Because included studies could be indexed in more than one database, the database coverage of each included study is summarized in Supplementary Table S1.

### 3.2 Characteristics of included studies

This study included 13 experimental studies published between 2006 and 2025, covering 318 DN animals. Among them, two studies used db/db mice (36/318, 11.32%), seven studies used Sprague-Dawley rats (210/318, 66.04%), three studies used Wistar rats (36/318, 11.32%), and one study used C57BL/6 mice (36/318, 11.32%). Regarding animal sex, 11 studies used male animals, while two studies used a mix of both sexes.

Three studies reported animal age, while 10 did not provide this information. Ten studies recorded the initial body weight of the animals, while three studies did not. Regarding the modeling method, eight studies used streptozotocin (STZ) to induce diabetes, two used spontaneous diabetic models, and three used alloxan for induction.

The blood glucose thresholds for successful modeling varied across studies, with four studies using a fasting glucose >16.6 mmol/L, one using >13.88 mmol/L, three studies using >200 mg/dL, and two studies using >250 mg/dL. One study did not specify the threshold. Intervention durations ranged from 4 to 12 weeks, with rutin doses ranging from 10 to 200 mg/kg. All 13 studies administered rutin via oral gavage.

Regarding outcome measures, all studies measured Scr, 11 studies measured BUN, seven studies reported 24-h UTP, and 11 studies measured blood glucose (Glu). Additionally, two studies evaluated triglycerides (TG) and total cholesterol (TC). Some studies assessed oxidative stress markers, including reactive oxygen species (ROS), superoxide dismutase (SOD), malondialdehyde (MDA), glutathione (GSH), and catalase (CAT). Moreover, some studies reported fibrosis-related signaling molecules, such as transforming growth factor-β (TGF-β). A detailed summary is provided in Table 1.

**TABLE 1:** Basic characteristics of the included studies.

Study(year)	Species; sex; age; weight	Model (number; establish; modeling standard)	Treatment group (rutin) (dose; number; administration; duration)	Outcome index	Intergroup differences
Wu et al. (2024)	db/db mice; male; 8 weeks; 40–45 g	n = 6; spontaneous diabetic model	Rutin-L: 30 mg/kg; n = 6; by Intragastric; 8weeks	Scr	*P* > 0.05
				BUN	*P* < 0.01
				UACR	*P* < 0.05
				Glu	*P* < 0.05
				TC	*P* > 0.05
				TG	*P* < 0.05
				ROS	*P* < 0.01
				SOD	*P* > 0.05
			Rutin-H: 100 mg/kg; n = 6; by Intragastric; 8weeks	Scr	*P* < 0.001
				BUN	*P* < 0.001
				UACR	*P* < 0.001
				Glu	*P* < 0.001
				TC	*P* < 0.001
				TG	*P* < 0.001
				ROS	*P* < 0.001
				SOD	*P* < 0.01
Manasa and Suhasin (2023)	SD rats; male; NR; NR	n = 6; Intraperitoneal injection of STZ (55 mg/kg); Glu >200 mg/dL	Rutin-L: 50 mg/kg; n = 6; by Intragastric; 8weeks	Scr	*P* < 0.05
				BUN	*P* < 0.05
				Glu	*P* < 0.05
				mALB	*P* < 0.05
				24-h UTP	*P* < 0.05
			Rutin-H: 100 mg/kg; n = 6; by Intragastric; 8weeks	Scr	*P* < 0.001
				BUN	*P* < 0.001
				Glu	*P* < 0.0001
				mALB	*P* < 0.01
				24-h UTP	*P* < 0.01
Dong et al. (2023)	db/db mice; male; 8 weeks; NR	n = 6; spontaneous diabetic model	Rutin-L: 100 mg/kg; n = 6; by Intragastric; 8weeks	Scr	*P* < 0.001
				BUN	*P* < 0.001
				mALB	*P* < 0.001
				UA	*P* < 0.05
				kidney index	*P* < 0.05
			Rutin-H: 200 mg/kg; n = 6; by Intragastric; 8weeks	Scr	*P* < 0.001
				BUN	*P* < 0.001
				mALB	*P* < 0.001
				UA	*P* < 0.001
				kidney index	*P* < 0.001
Zaghloul et al. (2022)	SD rats; male; NR; 200–220 g	n = 6; Intraperitoneal injection of STZ (45 mg/kg); Glu >250 mg/dL	Rutin: 100 mg/kg; n = 6; by Intragastric; 8weeks	Scr	*P* > 0.05
				BUN	*P* > 0.05
				24-h UTP	*P* > 0.05
				Glu	*P* < 0.05
				MDA	*P* < 0.05
				GSH	*P* < 0.05
				TNF-α	*P* > 0.05
				NF-κB	*P* < 0.05
				IL-6	*P* < 0.05
Gong et al. (2020)	C57BL/6 mice; male; NR; 35 ± 10 g	n = 12; Intraperitoneal injection of STZ (62.5 mg/kg); Glu >16.6 mmol/L	Rutin-L: 50 mg/kg; n = 12; by Intragastric; 8weeks	Scr	*P* < 0.05
				BUN	*P* < 0.01
				Glu	*P* < 0.05
				SOD	*P* > 0.05
				MDA	*P* < 0.05
				TGF-β	*P* > 0.05
				GSH	*P* < 0.05
			Rutin-H: 100 mg/kg; n = 12; by Intragastric; 8weeks	Scr	*P* < 0.05
				BUN	*P* < 0.01
				Glu	*P* > 0.05
				SOD	*P* < 0.05
				MDA	*P* < 0.05
				TGF-β	*P* < 0.05
				GSH	*P* < 0.01
Ganesan et al. (2020)	Wistar rats; male; NR; 180–220 g	n = 6; Intraperitoneal injection of alloxan (150 mg/kg); NR	Rutin: 100 mg/kg; n = 6; by Intragastric; 4weeks	Scr	*P* < 0.05
				BUN	*P* < 0.05
				TC	*P* < 0.05
				TG	*P* < 0.05
				Glu	*P* < 0.05
				chloride	*P* < 0.05
				bicarbonate	*P* < 0.05
				Fn	*P* < 0.05
				TGF-β	*P* < 0.05
				Urinary (pH)	*P* < 0.05
				Urinary β-hydroxy butyrate	*P* < 0.05
Ganesan et al. (2018)	Wistar rats; male; NR; 180–220 g	n = 6; Intraperitoneal injection of alloxan (150 mg/kg); Glu ≥200 mg/dL	Rutin: 100 mg/kg; n = 6; by Intragastric; 6weeks	Scr	*P* < 0.05
				Glu	*P* < 0.05
				Urine Creatinine	*P* < 0.05
				Urine Urea	*P* < 0.05
				Serum Calcium	*P* < 0.05
JH et al. (2015)	SD rats; half male, half female; NR; 180–220 g	n = 10; Intraperitoneal injection of alloxan (70 mg/kg), three consecutive times; Glu >16. 7 mmol/L	Rutin-L: 100 mg/kg; n = 10; by Intragastric; 12weeks	Scr	*P* < 0.01
				BUN	*P* < 0.01
				24-h UTP	*P* < 0.01
				Glu	*P* > 0.05
				kidney index	*P* < 0.05
			Rutin-H: 200 mg/kg; n = 10; by Intragastric; 12weeks	Scr	*P* < 0.01
				BUN	*P* < 0.01
				24-h UTP	*P* < 0.01
				Glu	*P* < 0.01
				kidney index	*P* < 0.01
JH et al. (2014)	SD rats; half male, half female; NR; 190–230 g	n = 10; High-fat diet for 4 weeks, intraperitoneal STZ (25 mg/kg), followed by another 4 weeks of high-fat diet; Glu >16. 7 mmol/L	Rutin-L: 50 mg/kg; n = 10; by Intragastric; 8weeks	Scr	*P* < 0.05
				BUN	*P* > 0.05
				24-h UTP	*P* < 0.01
				TC	*P* < 0.05
				TG	*P* < 0.05
				LDL	*P* > 0.05
				HDL	*P* > 0.05
				Glu	*P* < 0.01
				AngII	*P* < 0.05
				VEGF	*P* < 0.05
			Rutin-M: 100 mg/kg; n = 10; by Intragastric; 8weeks	Scr	*P* < 0.01
				BUN	*P* < 0.01
				24-h UTP	*P* < 0.01
				TC	*P* < 0.05
				TG	*P* < 0.01
				LDL	*P* < 0.05
				HDL	*P* < 0.05
				Glu	*P* < 0.01
				AngII	*P* < 0.01
				VEGF	*P* < 0.01
			Rutin-H: 200 mg/kg; n = 10; by Intragastric; 8weeks	Scr	*P* < 0.01
				BUN	*P* < 0.01
				24-h UTP	*P* < 0.01
				TC	*P* < 0.01
				TG	*P* < 0.01
				LDL	*P* < 0.01
				HDL	*P* < 0.01
				Glu	*P* < 0.01
				AngII	*P* < 0.01
				VEGF	*P* < 0.01
Hui-hui et al. (2013)	SD rats; male; NR; 180–220 g	n = 12; Intraperitoneal injection of STZ (60 mg/kg); Glu >16.7 mmol/L	Rutin-L: 50 mg/kg; n = 12; by Intragastric; 12weeks	Scr	*P* < 0.01
				BUN	*P* < 0.05
				24-h UTP	*P* < 0.01
				Glu	*P* < 0.01
				kidney index	*P* < 0.05
				CAT	*P* < 0.01
				SOD	*P* < 0.05
				GSH	*P* < 0.01
				MDA	*P* < 0.01
			Rutin-H: 100 mg/kg; n = 12; by Intragastric; 12weeks	Scr	*P* < 0.01
				BUN	*P* < 0.01
				24-h UTP	*P* < 0.01
				Glu	*P* < 0.01
				kidney index	*P* < 0.01
				CAT	*P* < 0.01
				SOD	*P* < 0.01
				GSH	*P* < 0.01
				MDA	*P* < 0.01
Hao et al. (2012)	SD rats; male; NR; 180–220 g	n = 8; Intraperitoneal injection of STZ (60 mg/kg); Glu >250 mg/dL	Rutin-L: 10 mg/kg; n = 8; by Intragastric; 10weeks	Scr	*P* < 0.05
				BUN	*P* < 0.05
				24-h UTP	*P* < 0.05
				Glu	*P* < 0.05
				MDA	*P* < 0.05
				SOD	*P* < 0.05
				GSH	*P* < 0.05
				CAT	*P* < 0.05
				TGF-β	*P* < 0.05
				Coll IV	*P* < 0.05
				kidney index	*P* < 0.05
			Rutin-M: 30 mg/kg; n = 9; by Intragastric; 10weeks	Scr	*P* < 0.01
				BUN	*P* < 0.01
				24-h UTP	*P* < 0.01
				Glu	*P* < 0.01
				MDA	*P* < 0.01
				SOD	*P* < 0.01
				GSH	*P* < 0.01
				CAT	*P* < 0.01
				TGF-β	*P* < 0.01
				Coll IV	*P* < 0.01
				kidney index	*P* < 0.01
			Rutin-H: 90 mg/kg; n = 9; by Intragastric; 10weeks	Scr	*P* < 0.01
				BUN	*P* < 0.01
				24-h UTP	*P* < 0.01
				Glu	*P* < 0.01
				MDA	*P* < 0.01
				SOD	*P* < 0.01
				GSH	*P* < 0.01
				CAT	*P* < 0.01
				TGF-β	*P* < 0.01
				Coll IV	*P* < 0.01
				kidney index	*P* < 0.01
SQ et al. (2012)	SD rats; male; NR; 200 ± 10 g	n = 10; Intraperitoneal injection of STZ (60 mg/kg); Glu ≥13.88 mmol/L	Rutin-L: 10 mg/kg; n = 10; by Intragastric; 12weeks	Scr	*P* < 0.05
				BUN	*P* < 0.05
				24-h UTP	*P* < 0.05
				kidney index	*P* < 0.05
				Glu	*P* < 0.05
			Rutin-M: 30 mg/kg; n = 10; by Intragastric; 12weeks	Scr	*P* < 0.01
				BUN	*P* < 0.01
				24-h UTP	*P* < 0.01
				kidney index	*P* < 0.01
				Glu	*P* < 0.01
			Rutin-H: 90 mg/kg; n = 10; by Intragastric; 12weeks	Scr	*P* < 0.01
				BUN	*P* < 0.01
				24-h UTP	*P* < 0.01
				kidney index	*P* < 0.01
				Glu	*P* < 0.01
Alsaif (2009)	Wistar rats; male; 8 weeks; 180 ± 20 g	n = 6; Intraperitoneal injection of STZ (70 mg/kg); Glu >200 mg/dL	Rutin: 100 mg/kg; n = 6; by Intragastric; 5weeks	Scr	*P* > 0.05
				Glu	*P* < 0.05
				ROS	*P* < 0.001
				MDA	*P* < 0.001
				SOD	*P* < 0.05
				GSH	*P* < 0.05
				CAT	*P* > 0.05

Scr: Serum Creatinine; BUN: blood urea nitrogen; UACR: Urine Albumin-to-Creatinine Ratio; Glu: Glucose; TC: total cholesterol; TG: triglycerides; ROS: reactive oxygen species; SOD: superoxide dismutase; mALB: microalbuminuria; 24-h UTP: 24-h Urinary Total Protein; UA: uric acid; MDA: malondialdehyde; GSH: glutathione; TNF-α: Tumor Necrosis Factor-alpha; NF-κB: Nuclear Factor kappa-B; IL-6: Interleukin-6; TGF-β: Transforming Growth Factor-beta; Fn: Fibronectin; LDL: Low-Density Lipoprotein; HDL: High-Density Lipoprotein; Ang II: Angiotensin II; VEGF: vascular endothelial growth factor; CAT: catalase; Coll IV: Collagen Type IV; Kidney index: Kidney Weight Index.

### 3.3 Study quality

**FIGURE 3:**
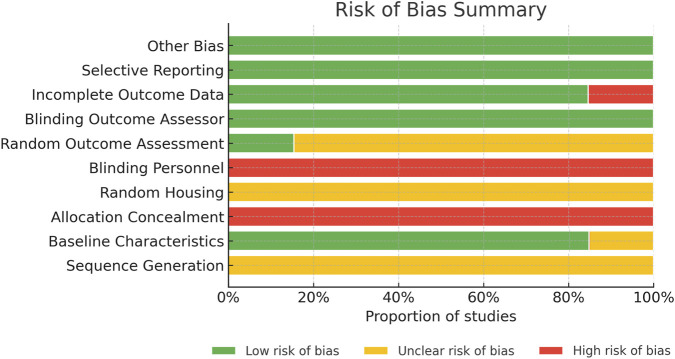
Risk of bias graph.

We evaluated the risk of bias of all included studies, as shown in Figure 3 and Supplementary Figure S1. The risk-of-bias scores of the included studies ranged from 4 to 6, indicating that several key items for bias control were incompletely reported. Although all 13 studies reported random allocation, none of them described the specific method used to generate the random sequence. Two studies did not provide information on baseline comparability. No study reported allocation concealment.

For performance bias, 12 studies were rated as high risk, with only one study assessed as low risk, indicating that blinding of personnel involved in the intervention was largely absent. In contrast, blinding of outcome assessment was generally adequate, suggesting that outcome evaluation was mostly conducted independently of treatment information. All studies reported outcomes consistent with their stated protocols, and no additional sources of bias were identified. In the supplementary section, we present the results of the stratified analysis, where studies were categorized into high-risk, medium-risk, and low-risk of bias based on the SYRCLE tool. The pooled effect sizes for each group are presented in the following figures (Supplementary Figures S2–S4; Supplementary Table S2). Overall, our analysis did not reveal significant differences in effect sizes between high-risk and low-risk groups (*P* > 0.05). However, a significant difference was observed between medium-risk and low-risk groups (*P* < 0.05), suggesting that the risk of bias may have a minor influence on effect sizes in these groups.

### 3.4 Effectiveness

#### 3.4.1 Primary outcomes

**FIGURE 4:**
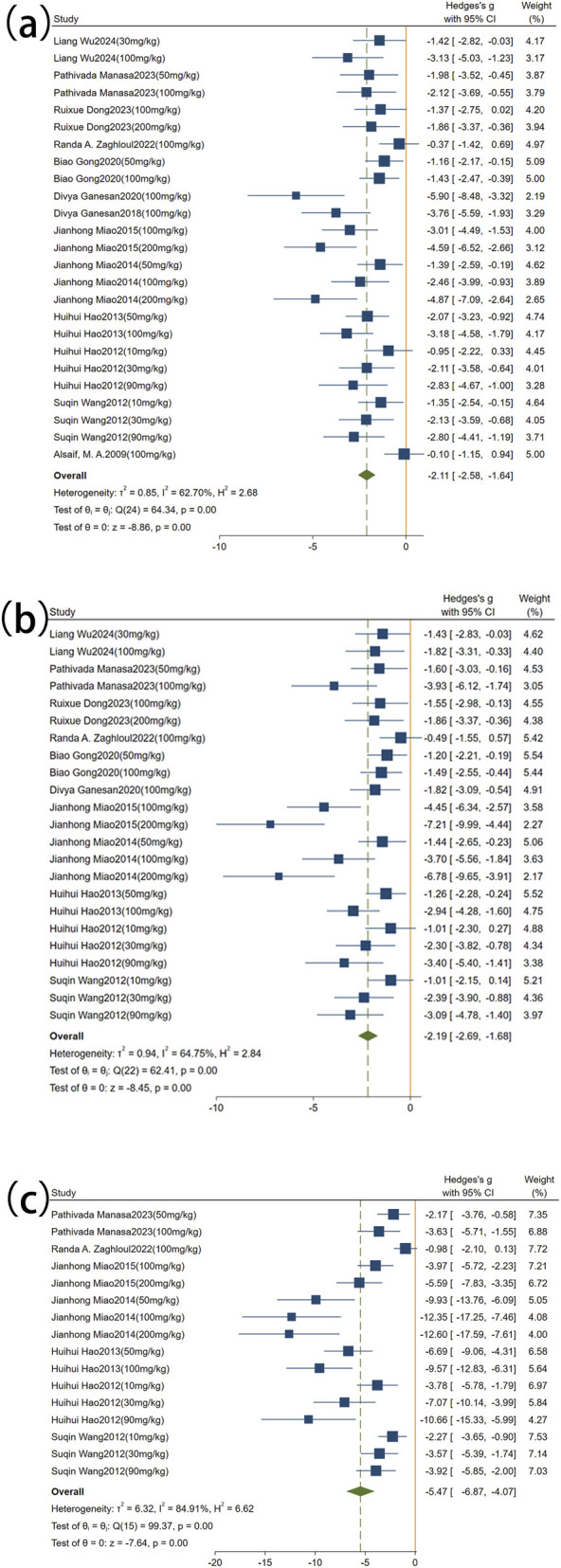
Forest plot: effect of rutin on renal function–related indicators. **(a)** Scr, **(b)** BUN, **(c)** 24-h UTP.

A total of 13 studies comprising 25 comparisons were included to evaluate the effect of rutin on Scr. The pooled results indicated that rutin significantly reduced Scr levels compared with the model group (SMD = −2.11, 95% CI: −2.58 to −1.64, *P* < 0.001), with moderate heterogeneity (I^2^ = 62.70%, *P* < 0.01; Figure 4a). For BUN, 11 studies with 23 comparisons were included. The pooled analysis showed that rutin significantly reduced BUN levels compared with the model group (SMD = −2.19, 95% CI: −2.69 to −1.68, *P* < 0.001), and moderate heterogeneity was observed (I^2^ = 64.75%, *P* < 0.01; Figure 4b). In addition, seven studies with 16 comparisons assessed the effect of rutin on 24-h UTP. The pooled results demonstrated that rutin significantly reduced 24-h UTP levels compared with the model group (SMD = −5.47, 95% CI: −6.87 to −4.07, *P* < 0.001), although high heterogeneity was observed (I^2^ = 84.91%, *P* < 0.01; Figure 4c).

#### 3.4.2 Secondary outcomes

**FIGURE 5:**
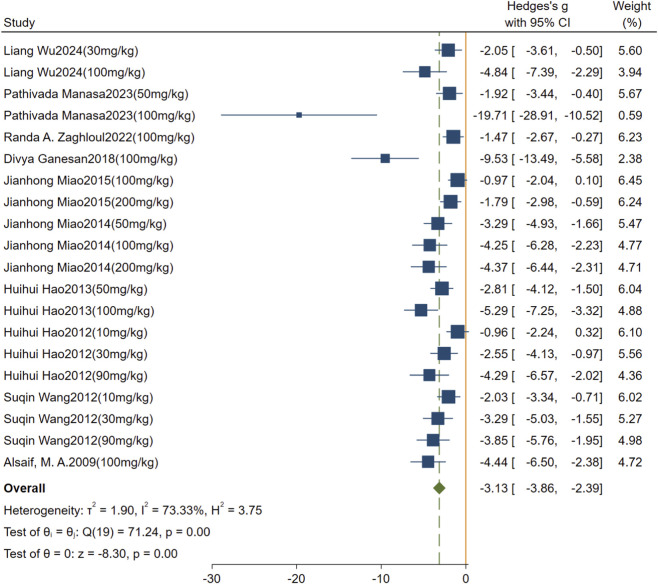
Forest plot: effect of rutin on Glu level.

Effect of rutin on blood Glu. A total of 11 studies comprising 20 comparisons were included. The pooled analysis showed that rutin significantly reduced Glu levels compared with the model group (SMD = −3.13, 95% CI: −3.86 to −2.39, *P* < 0.001). High heterogeneity was observed (I^2^ = 73.33%, *P* < 0.01); Figure 5.

**FIGURE 6:**
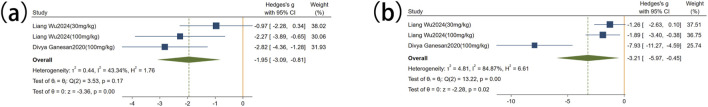
Forest plot: effect of rutin on lipid profile–related indicators. **(a)** TC, **(b)** TG.

Rutin exhibited significant lipid-lowering effects. Three studies provided data on TC levels. The results of a detailed analysis showed that, compared with the model group, rutin significantly reduced TC levels (n = 72, SMD = −1.95, 95% CI: −3.09 to −0.81, *P* < 0.05; heterogeneity: I^2^ = 43.34%, *P* = 0.17; Figure 6a). Moreover, three studies reported TG data, and the analysis demonstrated a significant reduction in TG levels following rutin treatment compared with the model group (n = 72, SMD = −3.21, 95% CI: −5.97 to −0.45, *P* < 0.05; heterogeneity: I^2^ = 84.87%, *P* < 0.01; Figure 6b). Overall, these results indicate that rutin exerts lipid-lowering effects in preclinical models.

**FIGURE 7:**
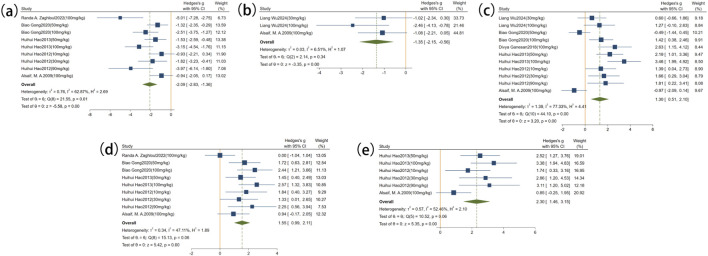
Forest plot: effect of rutin on oxidative stress–related indicators. **(a)** MDA, **(b)** ROS, **(c)** SOD, **(d)** GSH, **(e)** CAT.

Rutin was found to have considerable regulatory effects on oxidative stress biomarkers. There are five studies that gave the data regarding the MDA levels (Figure 7a). The analysis pooled demonstrated that rutin significantly decreased the level of MDA in comparison with the model control group (n = 113, SMD = −2.12, 95% CI: −2.83 to −1.41, *P* < 0.05; heterogeneity: I^2^ = 59.95%, *P* = 0.01). Also, three studies reported data on ROS (Figure 7b), and the results showed a significant decline in ROS levels in the rutin administration group in comparison with the model (n = 56, SMD = −1.78, 95% CI: −2.63 to −0.93, *P* < 0.05; heterogeneity: I^2^ = 6.71%, *P* = 0.34). Moreover, the data about SOD levels were on six studies (Figure 7c). In an overall assessment, it was found that rutin statistically increased the level of SOD as compared to the model (n = 132, SMD = 1.30, 95% CI: 0.51 to 2.10, *P* < 0.05; heterogeneity: I^2^ = 77.33%, *P* < 0.01). Five of them reported GSH levels (Figure 7d) with the result that rutin had a significant effect on GSH levels over the model group (n = 136, SMD = 1.63, 95% CI: 1.09 to 2.18, *P* < 0.05; heterogeneity: I^2^ = 43.14%, *P* = 0.08). Finally, three of the studies provided information about CAT levels (Figure 7e). The pooled analysis revealed a significant increase in CAT levels in rutin-treated animals compared with the model group (n = 102, SMD = 2.30, 95% CI: 1.46 to 3.15, *P* < 0.05; heterogeneity: I^2^ = 52.46%, *P* = 0.06). Combined, these data suggest that rutin has strong antioxidant activity in preclinical investigations.

**FIGURE 8:**
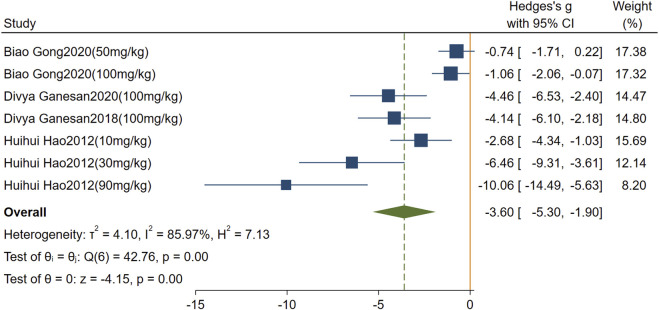
Forest plot: impact of rutin on TGF-β level.

Effect of rutin on TGF-β. A total of four studies comprising seven comparisons were included. The pooled analysis demonstrated that rutin significantly reduced TGF-β levels compared with the model group (SMD = −3.60, 95% CI: 5.30 to −1.90, *P* < 0.001). High heterogeneity was observed (I^2^ = 85.97%, p < 0.01); Figure 8.

### 3.5 Sensitivity analysis

Leave-one-out sensitivity analyses for Scr, BUN, and 24-h UTP showed that removing any single study did not materially change the pooled effect sizes. For Scr, the pooled Hedges’s g remained approximately between −2.19 and −2.00; for BUN, between −2.27 and −2.02; and for 24-h UTP, between −5.78 and −5.12. All estimates remained statistically significant with *P* < 0.001. These results indicate that no individual study unduly influenced the overall findings, confirming the robustness and stability of the meta-analytic estimates (Supplementary Figures S5–S7).

### 3.6 Subgroup analysis

Subgroup analyses were conducted for 24-h UTP, BUN, and Scr to explore potential sources of heterogeneity. Overall, rutin consistently improved renal outcomes across all subgroup categories. For 24-h UTP, stratification by intervention duration, dosage, animal species, and modeling method revealed comparable effect sizes, and no subgroup differences reached statistical significance (all *P* > 0.05), indicating that the reduction in proteinuria was stable across study characteristics. For BUN, several subgroup comparisons—particularly those based on duration and dosage—showed significant between-group differences (*P* < 0.05), suggesting that the extent of BUN reduction may be partly influenced by exposure time or dosing level. For Scr, a few subgroup tests yielded borderline or modestly significant differences, yet all subgroups maintained the same direction of effect, and none contradicted the overall finding. Although heterogeneity within many subgroups remained high (often I^2^ > 50%), the consistently beneficial direction across all subgroup categories supports the robustness of rutin’s renoprotective effects (Supplementary Figures S8–S19).

### 3.7 Publication bias

**FIGURE 9:**
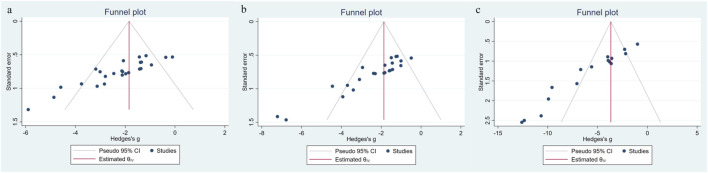
Funnel plot for **(a)** Scr, **(b)** BUN, **(c)** 24-h UTP.

**FIGURE 10:**
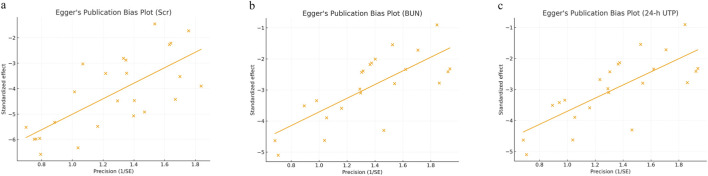
Eggerʼs publication bias plot for **(a)** Scr, **(b)** BUN, **(c)** 24-h UTP.

**FIGURE 11:**
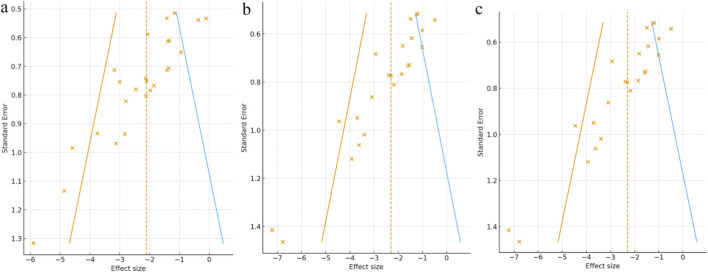
Trim-and-fill analysis for **(a)** Scr, **(b)** BUN, **(c)** 24-h UTP.

Potential publication bias was evaluated using funnel plots (Figure 9), Egger’s regression tests (Figure 10), and the Duval and Tweedie trim-and-fill method (Figure 11) for the three primary outcomes (Scr, BUN, and 24-h UTP). The funnel plots exhibited varying degrees of asymmetry, suggesting the presence of small-study effects. Egger’s tests further supported this observation, yielding statistically significant intercepts across all outcomes, indicating directional rather than random scatter of study estimates. To assess the potential impact of missing studies, the trim-and-fill procedure was applied as an exploratory sensitivity analysis. Although a small number of studies were imputed, the adjusted pooled effect sizes remained largely unchanged relative to the original estimates. However, trim-and-fill relies on assumptions that may not fully account for consistent small-study effects; therefore, these results should be interpreted cautiously and do not rule out publication or other reporting-related biases. Overall, under the trim-and-fill assumption, the direction and magnitude of the pooled effects were broadly similar, but our conclusions remain conservative.

### 3.8 Dose–time–response visualization

**FIGURE 12:**
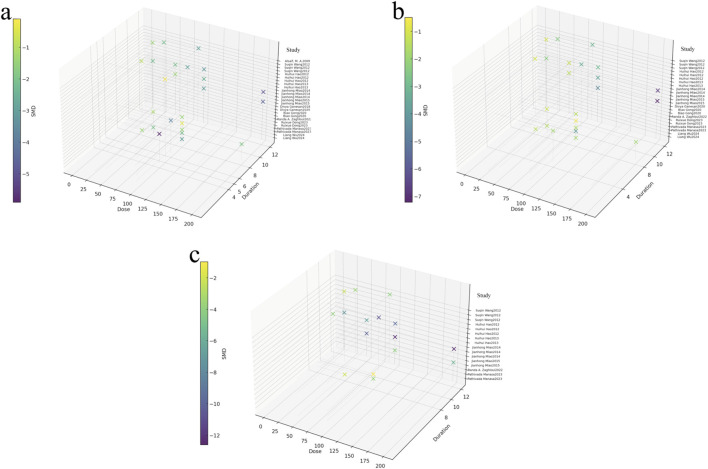
Three-dimensional dose–time–response relationships of rutin on key renal outcomes in DN. **(a)** Scr, **(b)** BUN, **(c)** 24-h UTP.

To describe the dose–time–response trends of rutin, 3D plots were generated for Scr (Figure 12a), BUN (Figure 12b), and 24-h UTP (Figure 12c). Across studies, effective data points were mainly concentrated within 25–200 mg/kg and 4–8 weeks. For Scr, most observable improvements clustered around 30–100 mg/kg and 6–8 weeks, indicating that moderate doses with mid-term treatment were more frequently associated with better outcomes. For BUN, responsive points showed a similar pattern, predominantly distributed between 25 and 100 mg/kg, with effective durations mainly around 4–8 weeks. For 24-h UTP, the effective region appeared slightly broader in dose, extending approximately from 20 to 150 mg/kg, but treatment durations remained mostly within 4–8 weeks. Overall, rutin demonstrated a relatively consistent therapeutic window, with most beneficial effects occurring at moderate doses (25–100 mg/kg) and short-to-mid treatment durations (four to eight weeks) rather than across the entire dose–time spectrum. We also estimated the human equivalent dose for the most frequently “effective” preclinical range (25–100 mg/kg) using standard body-surface-area scaling. This corresponds approximately to 4.1–16.2 mg/kg (rat-based) or 2.0–8.1 mg/kg (mouse-based), provided for context only rather than as a clinical dosing recommendation.

## 4 Discussion

### 4.1 Summary of evidence

The preclinical evidence on the use of rutin in animal models of DN has been summarized in this systematic review and meta-analysis. Thirteen studies involving 318 animals were included. Overall, rutin consistently improved renal function across different species and modeling strategies. It reduced Scr, BUN, and 24-h UTP. Beyond these renal indicators, rutin also relieved hyperglycemia, lipid abnormalities, oxidative stress, inflammation, and tissue fibrosis—suggesting that it acts on multiple pathological pathways in DN and may serve as a useful therapeutic adjunct.

Although the direction of effect was generally uniform, considerable heterogeneity existed among studies. This variation could be due to differences in animal species, model induction techniques, dose, treatment period, and measurement methods. Subgroup analyses showed that rutin exerted protective effects across nearly all categories, without reversing trends. The variation observed in BUN and Scr may relate to inconsistent doses and intervention length across studies. Publication bias was suggested by funnel plots and Egger’s test. After applying the trim-and-fill method, the pooled results remained largely unchanged, supporting the robustness of the findings. Our three-dimensional dose–time visualization indicated that most effective data were clustered around 25–100 mg/kg for 4–8 weeks, implying a relatively concentrated therapeutic range for rutin.

### 4.2 Mechanistic analysis

**FIGURE 13:**
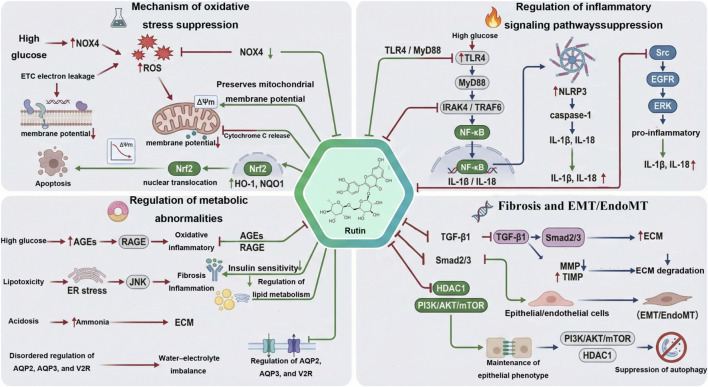
Mechanistic network of rutin in DN.

Integrating our meta-analysis with existing mechanistic evidence suggests that rutin protects the kidney through a network of actions rather than a single target. Its benefits arise from coordinated effects on oxidative stress, inflammation, fibrosis, and metabolic disturbance, which together slow the pathological progression of DN (Figure 13). To avoid overstating mechanistic certainty, we distinguish pathways directly supported by pooled quantitative outcomes from those discussed as narrative, hypothesis-generating interpretations based on individual experiments. In this meta-analysis, pooled data support mechanistic domains related to metabolic regulation, oxidative stress/antioxidant defense, and fibrosis-related signaling. In contrast, inflammatory pathways were not quantitatively synthesized because inflammatory outcomes were insufficiently and inconsistently reported across the included studies; therefore, inflammation-related signaling is discussed only to provide biological context. In addition, since blood glucose was also reduced in pooled analyses, the observed renal benefits may reflect both kidney-specific actions and indirect improvements related to metabolic control.

#### 4.2.1 Antioxidant

Oxidative stress is at the top of the pathogenesis system of DN, and its formation involves multiple mutually reinforcing signal circuits (Tanase et al., 2022; Ho and Shirakawa, 2022; Hu et al., 2021). Long-term high glucose exposure first enhances the expression of NADPH oxidase, especially NOX4, significantly increasing the oxidative load of the kidneys under basal conditions. Meanwhile, under high-glucose conditions, increased electron leakage from the mitochondrial electron transport chain leads to enhanced production of reactive oxygen species (Tanase et al., 2022; Ho and Shirakawa, 2022). It is well known that mitochondrial oxidative stress not only causes direct damage to membrane lipids, proteins, and nucleic acids, but also impairs mitochondrial membrane potential and oxidative phosphorylation efficiency, and promotes the release of cytochrome c into the cytoplasm, thereby triggering apoptotic cascade reactions (Ho and Shirakawa, 2022). On the other hand, the combination of AGE and RAGE can also continuously amplify oxidative stress through protein kinase C and various mitogen-activated kinases, gradually forming a three-dimensional oxidative stress network (Tanase et al., 2022; Cepas et al., 2020).

The function of rutin is not merely manifested as free radical scavenging, but rather it simultaneously intervenes in the source of oxidation and the oxidation response mechanism (Kamalakkannan and Prince, 2006; Rahmani et al., 2022). In various diabetes models, rutin can inhibit the expression of NOX4, thereby reducing the initial oxidative load of the kidneys. Its protection of mitochondrial function is of even more profound significance, including maintaining the stability of membrane potential, reducing the release of cytochrome c, and improving the energy metabolism state, thereby blocking the apoptosis and inflammatory amplification effects caused by mitochondrial dysfunction (Gong et al., 2020; Ganesan et al., 2020; Ganesan et al., 2018; Hao et al., 2012; Wang et al., 2017; Li et al., 2014; Jin and Zhang, 2024). On the other hand, rutin can promote the nuclear translocation of Nrf2, increase the expression of antioxidant genes such as HO-1 and NQO1, and enhance the endogenous antioxidant capacity of cells (Tabolacci et al., 2023; Zaghloul et al., 2022; Kamalakkannan and Prince, 2006). This bidirectional regulation of oxidation sources and oxidation responses enables rutin to effectively limit the persistent spread of oxidative stress in the early stage of DN, laying the foundation for blocking subsequent inflammation and fibrosis (Zaghloul et al., 2022; Tanase et al., 2022; Kamalakkannan and Prince, 2006).

#### 4.2.2 Anti-inflammatory

Under the background of accumulated oxidative stress, a complex inflammatory microenvironment rapidly forms locally in the kidneys, among which the signaling module centered on the TLR4-myD88-NF -κB pathway is the most crucial (Donate-Correa et al., 2020; Donate-Correa et al., 2021; Liu et al., 2014; Gholami Chahkand et al., 2024). Under high glucose stimulation, renal cells upregulate TLR4, and its downstream adaptor protein MyD88 then recruits and activates IRAK4 and TRAF6. This process eventually leads to NF-κB p65 entering the nucleus, triggering the transcription of various pro-inflammatory mediators (Liu et al., 2014; Gholami Chahkand et al., 2024). Continuous NF-κB activation not only promotes the infiltration of monocytes and macrophages, but also causes local inflammation to be in a state of continuous intensification, accelerating the damage of glomerular and tubular cells (Donate-Correa et al., 2020; Donate-Correa et al., 2021).

Meanwhile, the NLRP3 inflammasome plays a pivotal role in the process of inflammatory amplification. The activation of NF-κB can induce the expression of NLRP3 and its related components, while mitochondrial oxidative damage and changes in ionic homeostasis provide additional signals for its activation, enabling the activation of inflammatory caspase and promoting the formation of mature structures of IL-1β and IL-18 (Tanase et al., 2022; Ho and Shirakawa, 2022; Chen et al., 2025; Jiao and Tian, 2025). The continuous operation of this inflammatory amplification circuit is an important driver of renal structural destruction (Chen et al., 2025; Jiao and Tian, 2025). In addition, high glucose can induce the activation of Src kinase and further promote ERK signaling through the reactivation of transmembrane receptor EGFR, making the expression of inflammatory factors more significant. This non-classical inflammatory pathway is particularly important in podocyte injury (Wu et al., 2024).

Rutin can simultaneously exert regulatory effects at multiple nodes of the aforementioned inflammatory system. It can inhibit the expression of TLR4 and MyD88, significantly reducing the nuclear translocation degree of NF-κB. By improving oxidative stress and mitochondrial function, rutin can significantly reduce the activation tendency of NLRP3, thereby limiting the production of mature IL-1β. In addition, rutin has the ability to inhibit Src, which inhibits the activation of EGFR and ERK induced by high glucose, thereby reducing the expression of inflammatory genes (Jang et al., 2025; Wu et al., 2024; Rahmani et al., 2022; Wang et al., 2017; Jin and Zhang, 2024; Qu et al., 2019; Ma et al., 2024). The inhibition of inflammatory pathways by rutin shows multi-level and multi-pathway characteristics and is an important component for delaying the progression of renal injury (Wu et al., 2024; Rahmani et al., 2022; Donate-Correa et al., 2020; Chen et al., 2025).

#### 4.2.3 Anti-fibrosis

In DN, the persistent presence of inflammation and oxidative stress eventually prompts the kidneys to enter the fibrotic stage, and the TGF-β 1-Smad signaling is the core driving axis of this process (Balakumar, 2024; Dadras et al., 2017; Li et al., 2019). The continuous increase of TGF-β1 leads to the phosphorylation of Smad2 and Smad3 and their entry into the nucleus, thereby promoting the expression of various extracellular matrix proteins such as collagen and fibronectin. Meanwhile, TGF-β1 also inhibits the MMP family and upregulates TIMP, hindering matrix degradation and further exacerbating tissue hardening. Long-term accumulation of ECM deposition leads to thickening of the glomerular basement membrane and interstitial dilation, which is the key pathological basis for irreversible functional decline (Balakumar, 2024; Dadras et al., 2017; Li et al., 2019).

Meanwhile, the alteration of cell phenotypes constitutes another important mechanism for the progression of fibrosis. Renal tubular epithelial cells are prone to epithelial-mesenchymal transition under the dual stimulation of inflammation and oxidative stress, which is manifested as a decrease in epithelial markers and an increase in myofibroblast-like markers. Endothelial cells may also undergo similar phenotypic transitions, adding a new source to the interstitial cell bank (Balakumar, 2024; Dadras et al., 2017; Li et al., 2019). Autophagy disorder is regarded as an important factor promoting these phenotypic transformations, and the mTOR-related pathway plays a key role in it (Dong et al., 2023).

Rutin can play an intervention role in multiple key links of fibrosis formation. It can reduce the expression of TGF-β1, decrease the activation of Smad2 and Smad3, and thereby downregulate the genes related to ECM synthesis. Rutin can also restore autophagy levels, inhibit HDAC1 and its upstream PI3K/AKT/mTOR signaling, maintain the homeostatic phenotype of epithelial and endothelial cells, and reduce their tendency to transform into stromal cells (Dong et al., 2023; Hao et al., 2012; Wang et al., 2016; Jash et al., 2024). For this reason, the restrictive effect of rutin on fibrosis at the tissue structure level and its regulation at the molecular signal level form a mutually reinforcing relationship, endowing it with the potential to intervene in the process of DN (Dong et al., 2023; Hao et al., 2012; Balakumar, 2024; Dadras et al., 2017; Li et al., 2019; Wang et al., 2016; Jash et al., 2024). At the same time, we acknowledge that the pooled reduction in TGF-β, while supportive of an antifibrotic association, may also partly reflect downstream effects secondary to broader metabolic and oxidative improvements.

#### 4.2.4 Metabolic regulation

Metabolic imbalance is the root factor of DN and is highly coupled with multiple pathological pathways such as oxidative stress, inflammation and fibrosis (Tanase et al., 2022; Hu et al., 2021; Efio et al., 2025). Hyperglycemia promotes the continuous generation of AGEs, and the combination of AGEs and RAGE activates various oxidative and inflammatory signals, forming a continuously enhanced positive feedback system of oxidative stress and inflammatory response (Cepas et al., 2020). Abnormal lipid metabolism is also involved in multiple pathological processes. Excessive lipids can induce endoplasmic reticulum stress and JNK signaling activation, thereby promoting inflammatory responses and TGF-β1-mediated fibrotic pathways (Efio et al., 2025). In addition, metabolic acidosis is a common but often overlooked pathological state in DN. It further promotes interstitial inflammation and ECM hyperplasia by increasing ammonia production and tissue acid load (Khairallah and Scialla, 2017).

The regulation of rutin at the metabolic level has systematic characteristics. It can improve insulin sensitivity and glucose utilization efficiency, and reduce the stimulation of high blood sugar on the kidneys from the source. By regulating lipid metabolism, the inflammatory and fibrotic signals related to lipid toxicity are weakened (Gong et al., 2020; Ganesan et al., 2020; Ganesan et al., 2018; Hao et al., 2012; Kamalakkannan and Prince, 2006; Singh et al., 2024). More importantly, rutin can reduce the formation of AGE and inhibit the expression of RAGE, effectively blocking the positive feedback loop between metabolism and inflammation (Singh et al., 2024; Xiang et al., 2021; Wu et al., 2021). In addition, the regulation of AQP2, AQP3 and V2R by rutin can improve the processing efficiency of water and electrolytes by renal tubules, and help alleviate acidosis and the interstitial damage it causes (Ganesan et al., 2020; Ganesan et al., 2018; Khairallah and Scialla, 2017). Therefore, the multi-dimensional regulation of rutin on metabolic abnormalities not only affects the early metabolic stress but also profoundly influences the process of downstream structural changes and functional decline (Ganesan et al., 2020; Ganesan et al., 2018; Efio et al., 2025; Khairallah and Scialla, 2017; Singh et al., 2024).

### 4.3 Limitations and future perspectives

This study systematically evaluated the preclinical evidence of rutin in the treatment of DN based on existing animal experiments, but there are still certain limitations. Firstly, the included studies showed significant differences in animal species, strains, modeling methods (STZ, alloxan, db/db), dose range (10–200 mg/kg), intervention duration (4–12 weeks), and experimental conditions. Such methodological and biological heterogeneity may affect the stability and generalizability of the effect size. Moreover, diagnostic criteria and glycemic thresholds for model establishment varied across studies, and baseline disease severity was often insufficiently reported, which may have reduced the comparability of outcomes and introduced residual confounding. Additionally, sex was insufficiently considered across studies. As most experiments used male animals, and sex-stratified outcomes were not reported, potential sex-specific responses to rutin could not be assessed. Secondly, only 13 studies and 318 animals were ultimately included, with a relatively small overall sample size. This may reduce the statistical power of the meta-analysis and increase the risk of publication bias, which may increase the likelihood of an overestimation of the therapeutic effect.

In addition, data from some studies were extracted from graphs using WebPlotDigitizer. Even after cross-validation by two researchers, there may still be minor errors. In terms of methodological quality, most of the literature fails to adequately report key information such as randomization, blinding, and allocation concealment, resulting in a high or unclear risk of bias. In particular, the lack of reported allocation concealment may have contributed to an overestimation of effect sizes and limits the internal validity of individual studies. Although our stratified analysis revealed no significant difference between high-risk and low-risk studies, the observed effect size differences between medium-risk and low-risk studies suggest that bias risk may have influenced some of the reported effects. Therefore, the pooled effect sizes in this meta-analysis should not be interpreted as precise or unbiased estimates of treatment efficacy, but rather as a quantitative summary of the overall direction and consistency of effects across studies. Accordingly, our conclusions should be interpreted cautiously, especially for outcomes with substantial heterogeneity. Nevertheless, the consistency of findings observed across multiple independent experiments and different animal models suggests that the observed associations are unlikely to be driven by a single study or methodological artifact. Finally, there are essential differences between animal models and human DN in terms of disease course characteristics, metabolic status and pharmacokinetics, which makes the convertibility of the results in clinical practice uncertain. Another translational limitation is that no included animal studies performed head-to-head or combination comparisons between rutin and established standard-of-care therapies for DN (e.g., ACEi/ARB or SGLT2 inhibitors); therefore, the relative efficacy and potential add-on benefit of rutin remain uncertain. Future studies should incorporate active-comparator and add-on designs with clinically relevant background therapy to strengthen translational interpretation.

More broadly, to further strengthen the preclinical evidence base, future research should adopt more standardized and rigorous experimental designs, expand sample sizes, and improve reporting transparency to strengthen the robustness and reproducibility of preclinical evidence. Greater attention to baseline disease severity, consideration of sex as a biological variable, and harmonization of outcome measures across DN models would further enhance translational relevance. In addition, systematic evaluation of dose–time relationships, together with basic pharmacokinetic and safety profiling, may help reduce uncertainty prior to clinical translation. Meanwhile, deeper mechanistic investigations using multi-omics approaches could further support the preclinical and clinical development of rutin.

## 5 Conclusion

This meta-analysis provides preclinical evidence supporting rutin as a potential therapeutic candidate for DN. Its multi-target effects on metabolic disturbances, oxidative stress, and fibrosis highlight its potential. These findings may help inform future standardized preclinical experiments and translational research. However, further standardized studies and clinical trials are needed to confirm its efficacy and therapeutic role in humans.

## Data Availability

The original contributions presented in the study are included in the article/Supplementary Material, further inquiries can be directed to the corresponding authors.
