# Supplementary material for: Effects of rutin on renal function, oxidative stress and fibrosis in animal models of diabetic nephropathy: a systematic review and meta-analysis
Source: Front Pharmacol. 2026 Feb 23;17:1771010. doi: 10.3389/fphar.2026.1771010 (PMC12968236; doi:10.3389/fphar.2026.1771010)
Supplement: Supplementary file 1 [file Supplementaryfile1.docx]

Supplementary Material

**search strategy**

**Pubmed：**

(

"diabetic nephropathies"[MeSH Terms]

OR ("diabetic"[All Fields] AND "nephropathies"[All Fields])

OR "diabetic nephropathies"[All Fields]

OR ("nephropathies"[All Fields] AND "diabetic"[All Fields])

OR "nephropathies diabetic"[All Fields]

OR ("nephropathy"[All Fields] AND "diabetic"[All Fields])

OR "nephropathy diabetic"[All Fields]

OR ("diabetic"[All Fields] AND "kidney"[All Fields] AND "disease"[All Fields])

OR "diabetic kidney disease"[All Fields]

OR ("diabetic"[All Fields] AND "kidney"[All Fields] AND "diseases"[All Fields])

OR "diabetic kidney diseases"[All Fields]

OR ("kidney"[All Fields] AND "disease"[All Fields] AND "diabetic"[All Fields])

OR "kidney disease diabetic"[All Fields]

OR ("kidney"[All Fields] AND "diseases"[All Fields] AND "diabetic"[All Fields])

OR "kidney diseases diabetic"[All Fields]

OR ("diabetic"[All Fields] AND "nephropathy"[All Fields])

OR "diabetic nephropathy"[All Fields]

OR ("diabetic"[All Fields] AND "glomerulosclerosis"[All Fields])

OR "diabetic glomerulosclerosis"[All Fields]

OR ("glomerulosclerosis"[All Fields] AND "diabetic"[All Fields])

OR "glomerulosclerosis diabetic"[All Fields]

OR ("intracapillary"[All Fields] AND "glomerulosclerosis"[All Fields])

OR "intracapillary glomerulosclerosis"[All Fields]

OR ("kimmelstiel"[All Fields] AND "wilson"[All Fields] AND "disease"[All Fields])

OR "kimmelstiel wilson disease"[All Fields]

OR ("kimmelstiel"[All Fields] AND "wilson"[All Fields] AND "syndrome"[All Fields])

OR "kimmelstiel wilson syndrome"[All Fields]

OR ("nodular"[All Fields] AND "glomerulosclerosis"[All Fields])

OR "nodular glomerulosclerosis"[All Fields]

OR ("glomerulosclerosis"[All Fields] AND "nodular"[All Fields])

OR "glomerulosclerosis nodular"[All Fields]

OR ("syndrome"[All Fields] AND "kimmelstiel"[All Fields] AND "wilson"[All Fields])

)

AND

(

"rutin"[Supplementary Concept]

OR "rutin"[All Fields]

OR "rutin"[MeSH Terms]

OR "rutins"[All Fields]

OR "rutoside"[All Fields]

OR "rutosides"[All Fields]

OR "quercetin 3 rutinoside"[All Fields]

)

**Embase**

(Nephropathies, Diabetic or Nephropathy, Diabetic or Diabetic Kidney Disease or Diabetic Kidney Diseases or Kidney Disease, Diabetic or Kidney Diseases, Diabetic or Diabetic Nephropathy or Diabetic Glomerulosclerosis or Glomerulosclerosis, Diabetic or Intracapillary Glomerulosclerosis or Kimmelstiel-Wilson Disease or Kimmelstiel Wilson Disease or Nodular Glomerulosclerosis or Glomerulosclerosis, Nodular or Kimmelstiel-Wilson Syndrome or Kimmelstiel Wilson Syndrome or Syndrome, Kimmelstiel-Wilson).af.

And

(Rutin or Rutoside or Quercetin-3-Rutinoside or Quercetin 3 Rutinoside or 3-Rhamnosyl-Glucosyl Quercetin or Quercetin, 3-Rhamnosyl-Glucosyl).af.

**Web of Science**

(

Diabetic Nephropathies (Topic) OR Nephropathies, Diabetic (Topic) OR Nephropathy, Diabetic (Topic)

OR Diabetic Kidney Disease (Topic) OR Diabetic Kidney Diseases (Topic) OR Kidney Disease, Diabetic (Topic)

OR Kidney Diseases, Diabetic (Topic) OR Diabetic Nephropathy (Topic) OR Diabetic Glomerulosclerosis (Topic)

OR Glomerulosclerosis, Diabetic (Topic) OR Intracapillary Glomerulosclerosis (Topic)

OR Kimmelstiel-Wilson Disease (Topic) OR Kimmelstiel Wilson Disease (Topic)

OR Nodular Glomerulosclerosis (Topic) OR Glomerulosclerosis, Nodular (Topic)

OR Kimmelstiel-Wilson Syndrome (Topic) OR Kimmelstiel Wilson Syndrome (Topic)

OR Syndrome, Kimmelstiel-Wilson (Topic)

)

AND

(

Rutin (Topic) OR Rutoside (Topic) OR Quercetin-3-Rutinoside (Topic)

OR Quercetin 3 Rutinoside (Topic) OR 3-Rhamnosyl-Glucosyl Quercetin (Topic)

OR Quercetin, 3-Rhamnosyl-Glucosyl (Topic)

)

AND

Preprint Citation Index (Exclude – Database)

AND

Research Commons (Exclude – Database)

**CNKI, Wangfang Data, VPCS:**

Search terms included: “芦丁” (rutin); “糖尿病肾病” (diabetic nephropathy).
Boolean operator AND was used to combine terms. No language restrictions were applied within Chinese databases.

**supplementary Table：**

**Supplementary Table 1. Database Sources of the Included Studies (n = 13)**

| **Included study** | **PubMed** | **Embase** | **Web of Science** | **CNKI** | **Wanfang** | **VIP** |
| --- | --- | --- | --- | --- | --- | --- |
| **Wu, 2024** | **✓** | **✓** | **✓** | **–** | **–** | **–** |
| **Manasa, 2023** | **–** | **✓** | **–** | **–** | **–** | **–** |
| **Dong, 2023** | **✓** | **✓** | **✓** | **–** | **–** | **–** |
| **Zaghloul, 2022** | **✓** | **✓** | **✓** | **–** | **–** | **–** |
| **Gong, 2020** | **–** | **✓** | **✓** | **–** | **–** | **–** |
| **Ganesan, 2020** | **✓** | **✓** | **✓** | **–** | **–** | **–** |
| **Ganesan, 2018** | **✓** | **✓** | **✓** | **–** | **–** | **–** |
| **Miao, 2015** | **–** | **–** | **–** | **✓** | **✓** | **✓** |
| **Miao, 2014** | **–** | **–** | **–** | **✓** | **–** | **–** |
| **Hao, 2013** | **–** | **–** | **–** | **✓** | **–** | **✓** |
| **Hao, 2012** | **✓** | **✓** | **✓** | **–** | **–** | **–** |
| **Wang, 2012** | **–** | **–** | **–** | **✓** | **✓** | **✓** |
| **Alsaif, 2009** | **–** | **✓** | **–** | **–** | **–** | **–** |

**Note: A check mark (✓) indicates that the included study is retrievable/indexed in the corresponding database based on the exported search results. Because many studies are indexed in multiple databases, one study may appear in more than one database.**

**Supplementary Table 2** Comparison of effect sizes (Hedges's g) and standard errors (SE) between high-risk, medium-risk, and low-risk studies based on the risk of bias assessment

| **Comparison Group** | **High-Risk**  **Effect Size** | **High-Risk (SE)** | **Medium-Risk Effect Size** | **Medium-Risk (SE)** | **Low-Risk Effect Size** | **Low-Risk (SE)** | **Z-Value** | **p-Value** |
| --- | --- | --- | --- | --- | --- | --- | --- | --- |
| **High-Risk vs Medium-Risk** | **-2.38** | **0.538** | **-1.79** | **0.206** |  |  | **-1.03** | **> 0.05** |
| **High-Risk vs Low-Risk** | **-2.38** | **0.538** |  |  | **-3.72** | **0.692** | **1.53** | **> 0.05** |
| **Medium-Risk vs Low-Risk** |  |  | **-1.79** | **0.206** | **-3.72** | **0.692** | **2.68** | **< 0.05** |

Statistical comparisons were conducted using Z-tests to assess whether high-risk-of-bias studies report systematically larger effects compared to lower-risk studies. The Z-values and p-values reflect the significance of the differences between the groups.

High-Risk vs Medium-Risk: No significant difference was found between the high-risk and medium-risk studies (Z = -1.03, p > 0.05).

High-Risk vs Low-Risk: No significant difference was observed between the high-risk and low-risk studies (Z = 1.53, p > 0.05).

Medium-Risk vs Low-Risk: A significant difference in effect sizes was found between medium-risk and low-risk studies, with low-risk studies reporting a larger effect size (Z = 2.68, p < 0.05).

**supplementary Figure：**

**
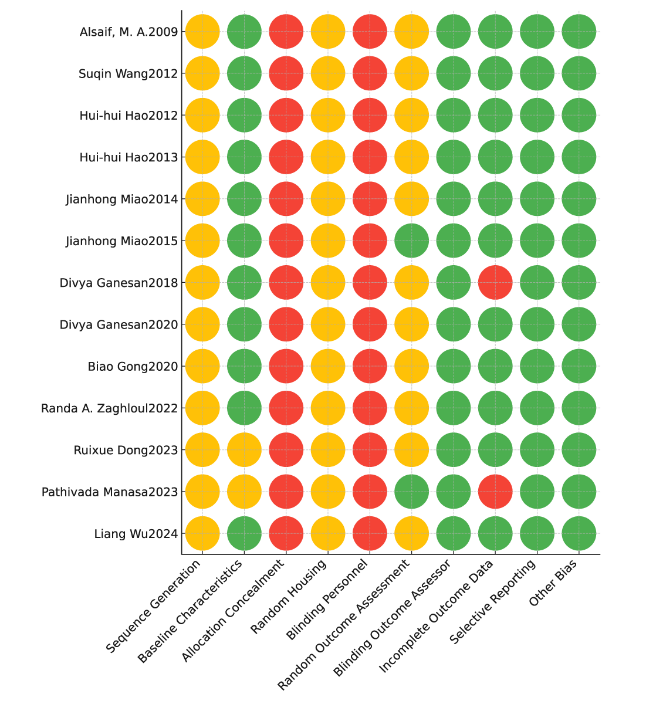
**

**supplementary Figure 1. Risk of bias summary**

**
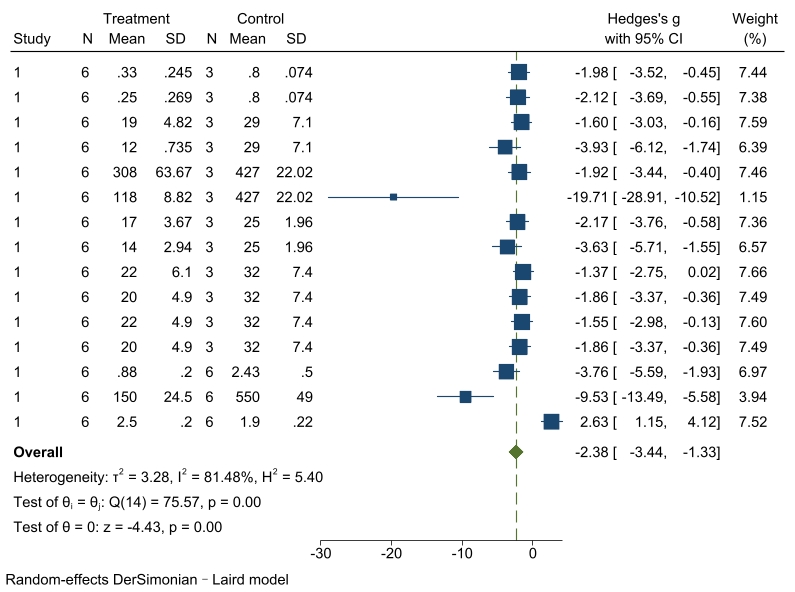
**

**supplementary Figure 2. Forest plot comparing the effect sizes (Hedges's g) of high-risk studies (4 points) in the treatment of diabetic nephropathy.**

**The plot shows the pooled effect size and 95% confidence interval (CI) for high-risk studies. The individual studies are marked, and the overall effect size is indicated by the diamond at the bottom of the plot.**

**
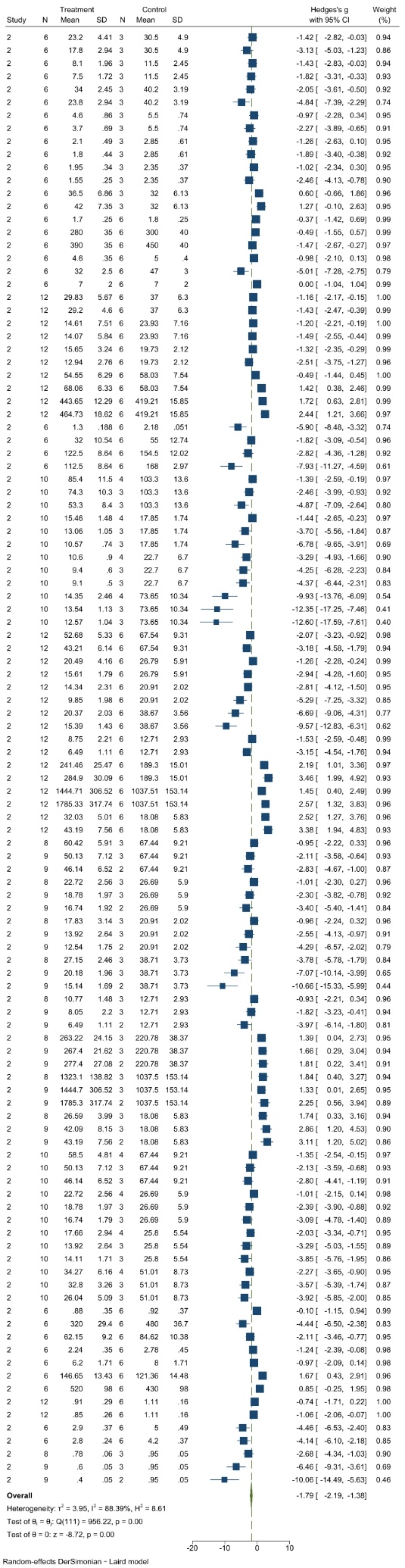
**

**supplementary Figure 3. Forest plot comparing the effect sizes (Hedges's g) of medium-risk studies (5 points) in the treatment of diabetic nephropathy. This plot presents the pooled effect size and 95% confidence interval (CI) for medium-risk studies. The individual study results and the overall effect size are shown for clarity.**

**
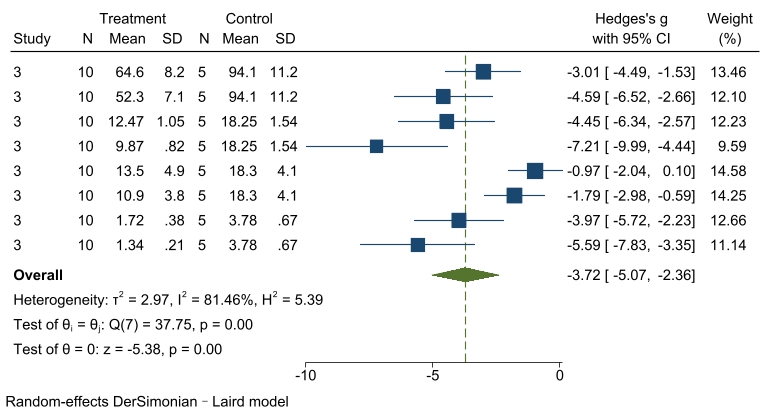
**

**supplementary Figure 4. Forest plot comparing the effect sizes (Hedges's g) of low-risk studies (6 points) in the treatment of diabetic nephropathy. The plot shows the pooled effect size with the corresponding 95% confidence interval (CI) for low-risk studies. Each individual study’s effect size is presented, with the diamond representing the overall pooled effect size**

**
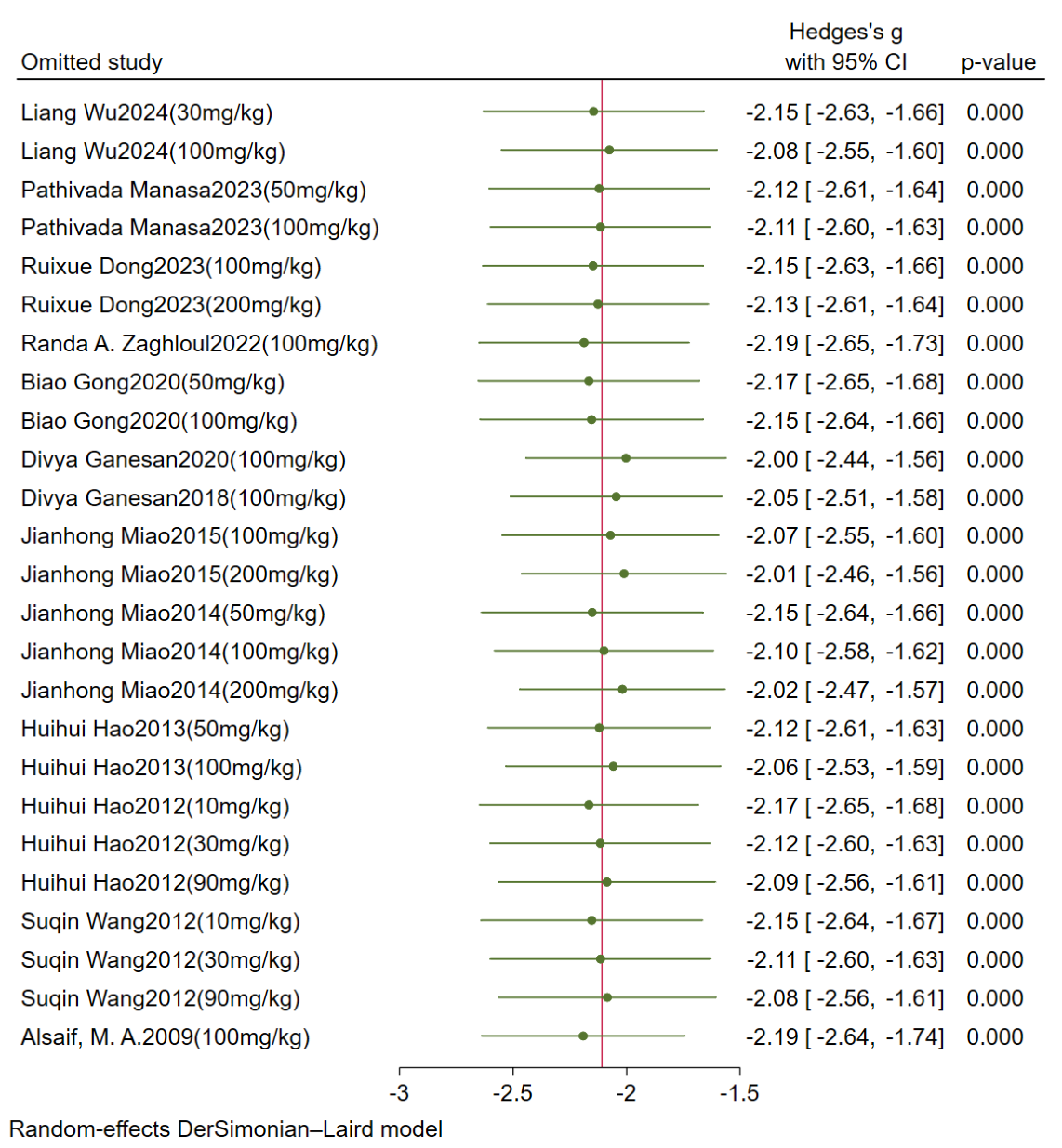
**

**Supplementary Figure 5. sensitivity analysis of SCR**

**
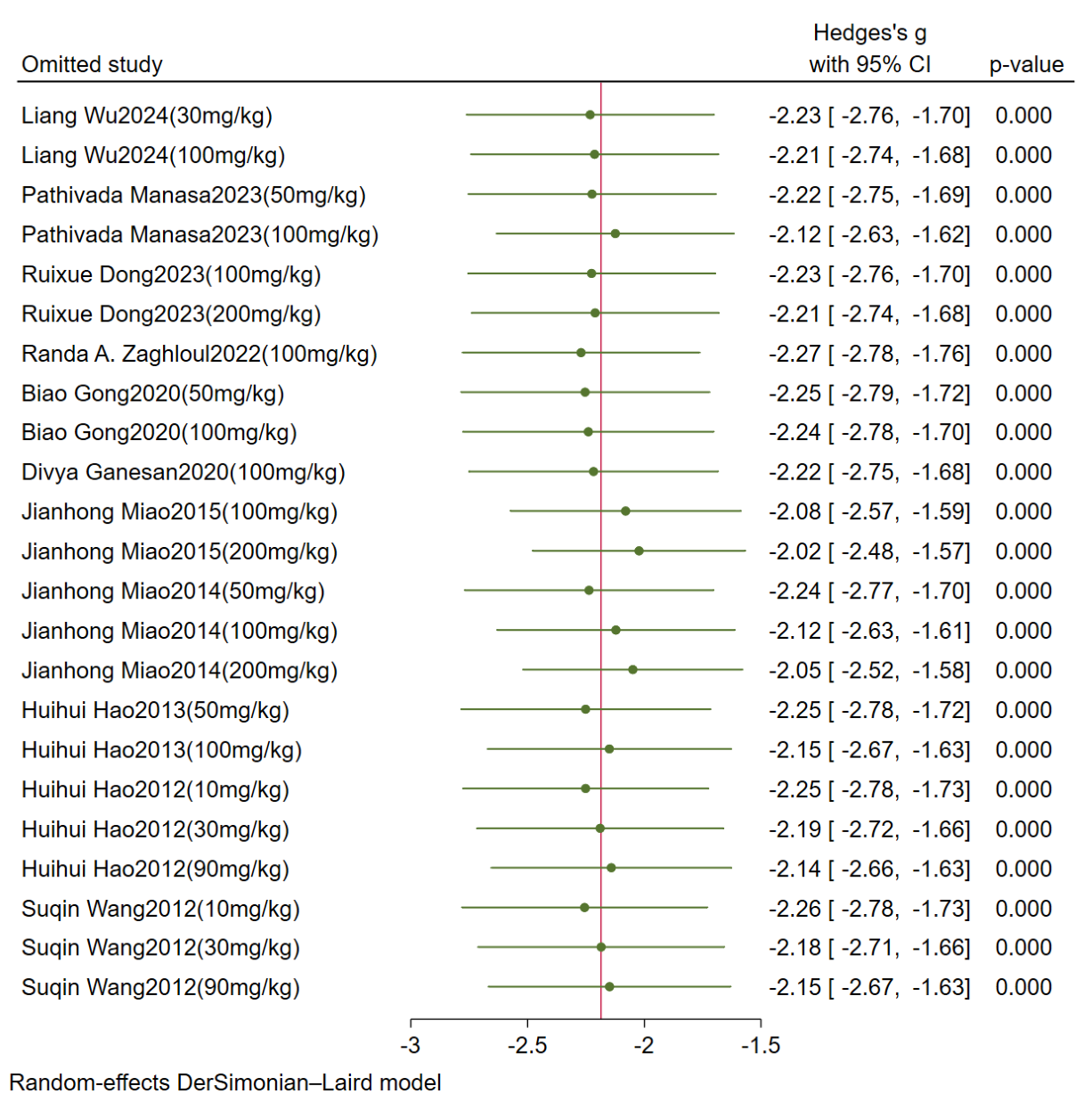
**

**Supplementary Figure 6. sensitivity analysis of BUN**

**
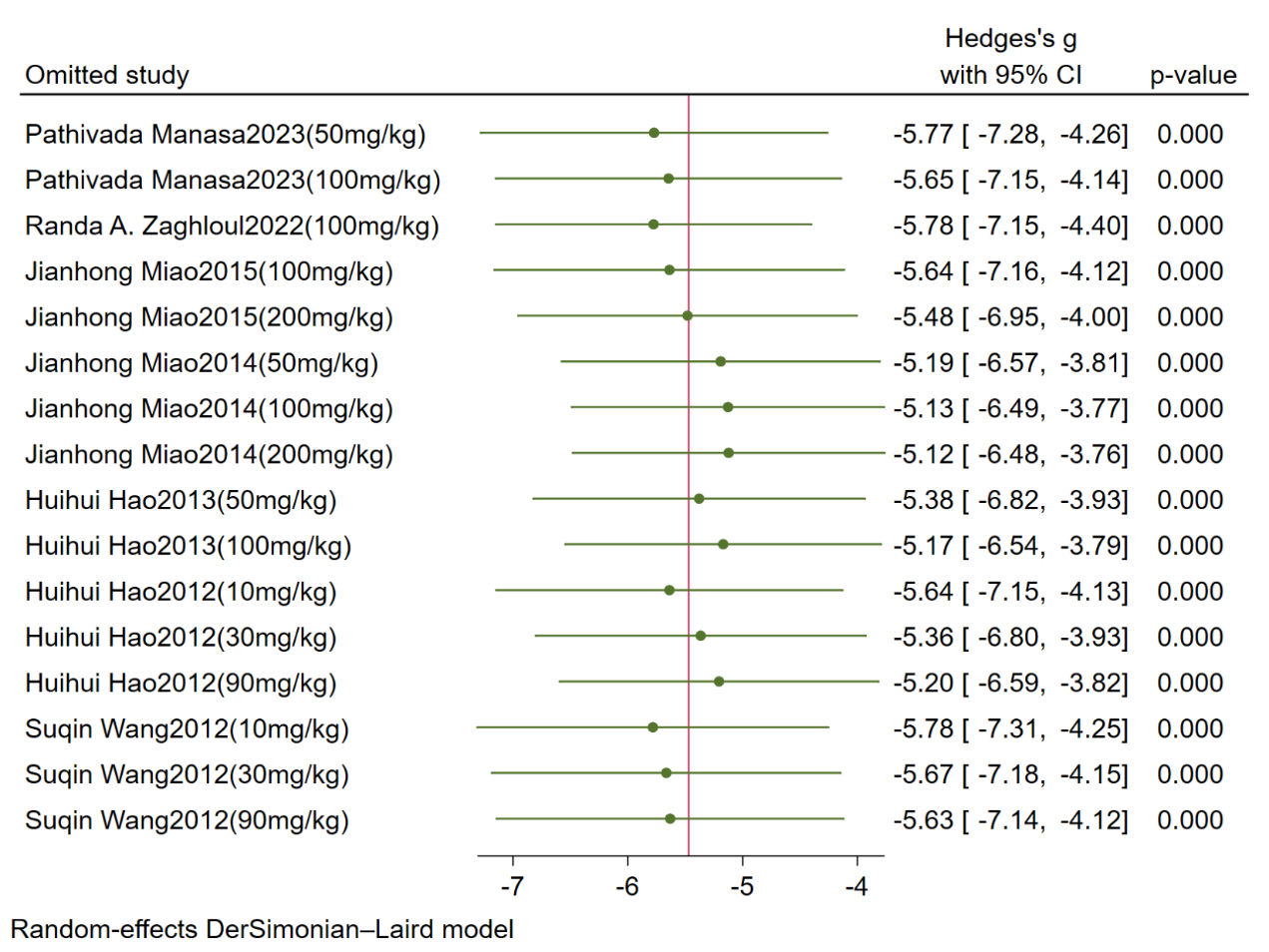
**

**Supplementary Figure 7. sensitivity analysis of 24-h UTP**

**
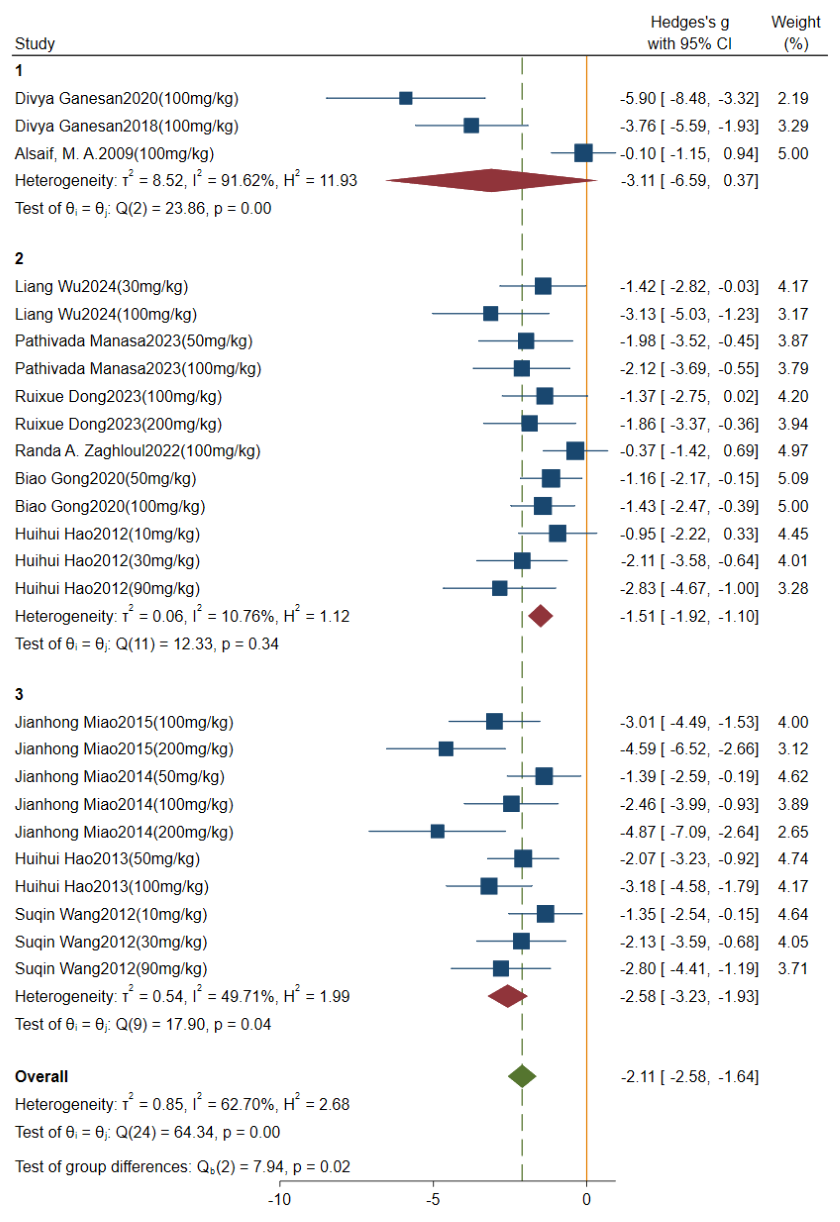
**

**Supplementary Figure 8. Subgroup analysis of Scr based on intervention duration**

**
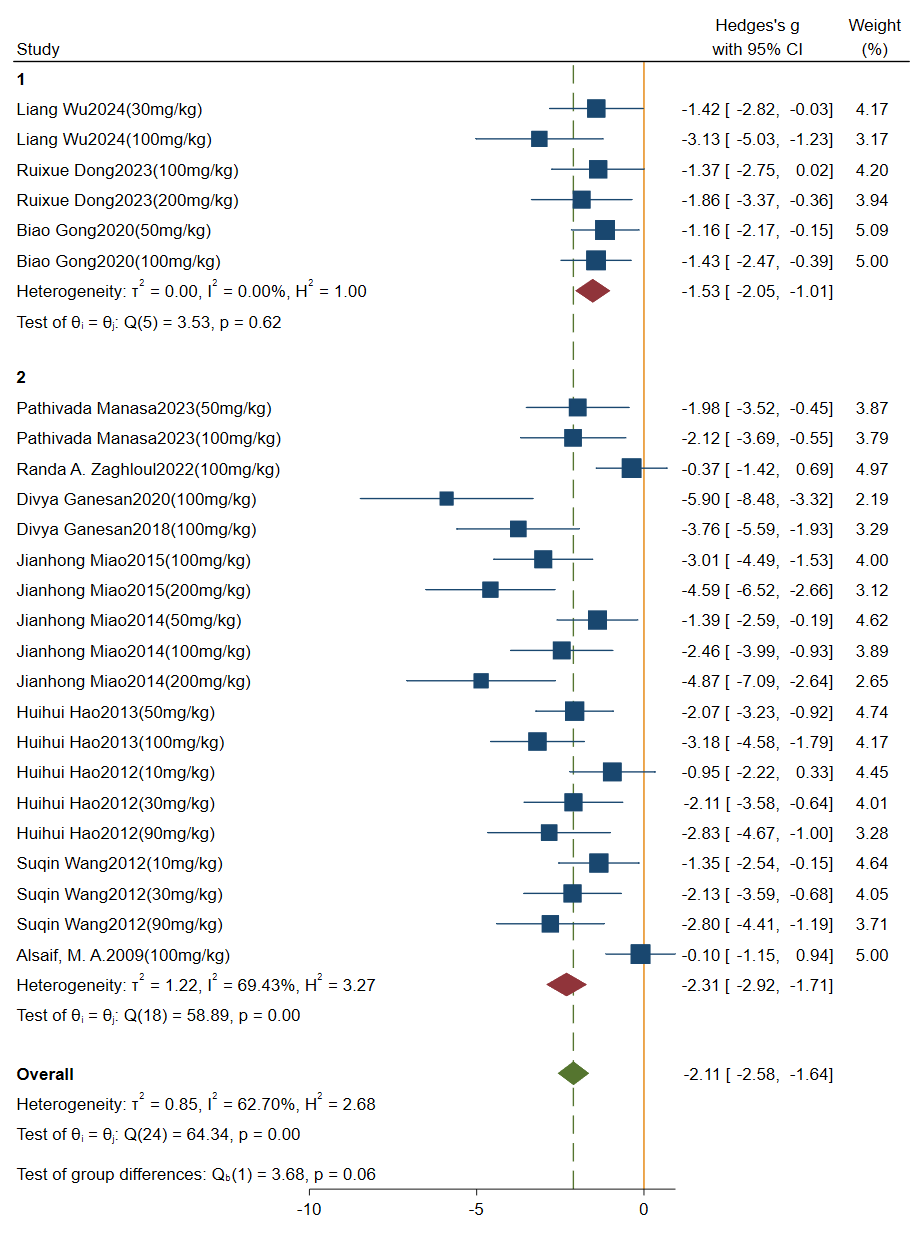
**

**Supplementary Figure 9. Subgroup analysis of Scr based on species of animals**

**
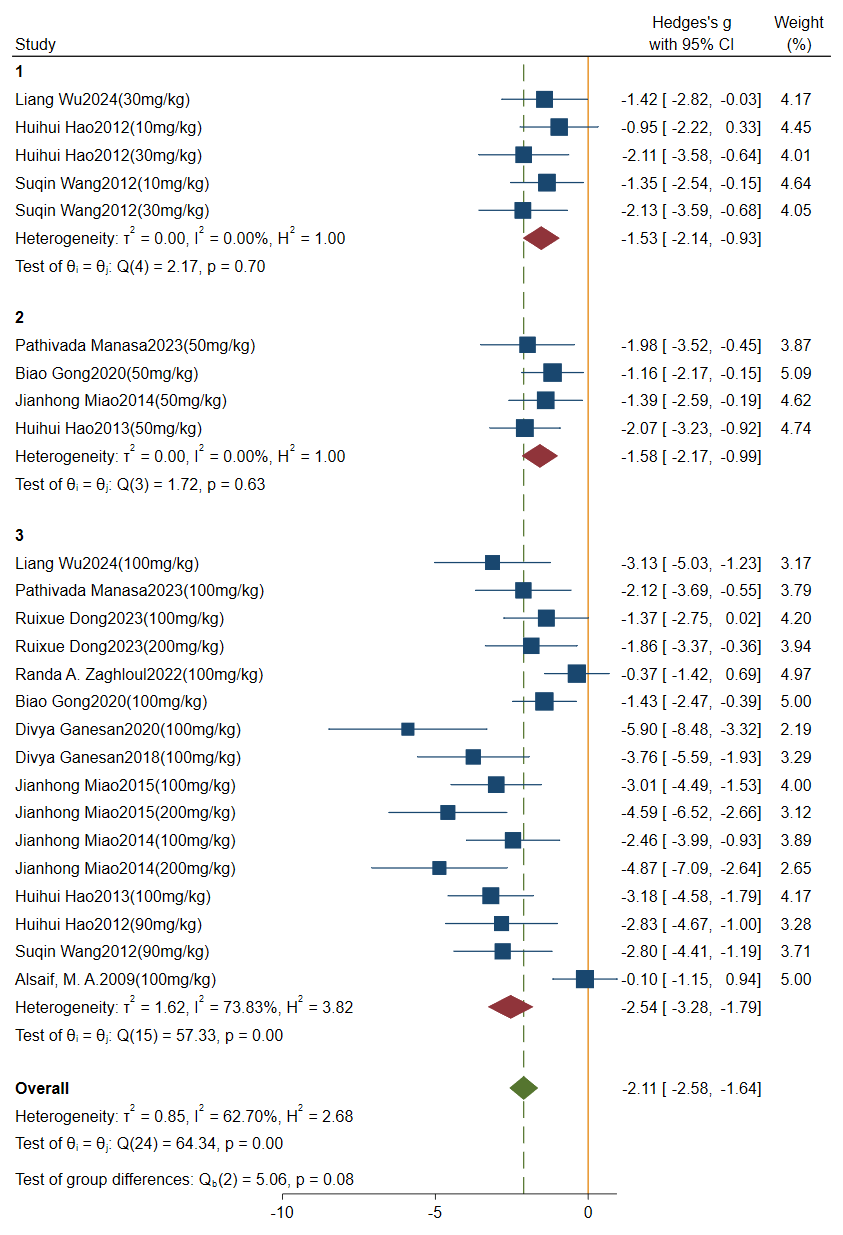
**

**Supplementary Figure 10. Subgroup analysis of Scr based on dosage regimens of rutin**

**
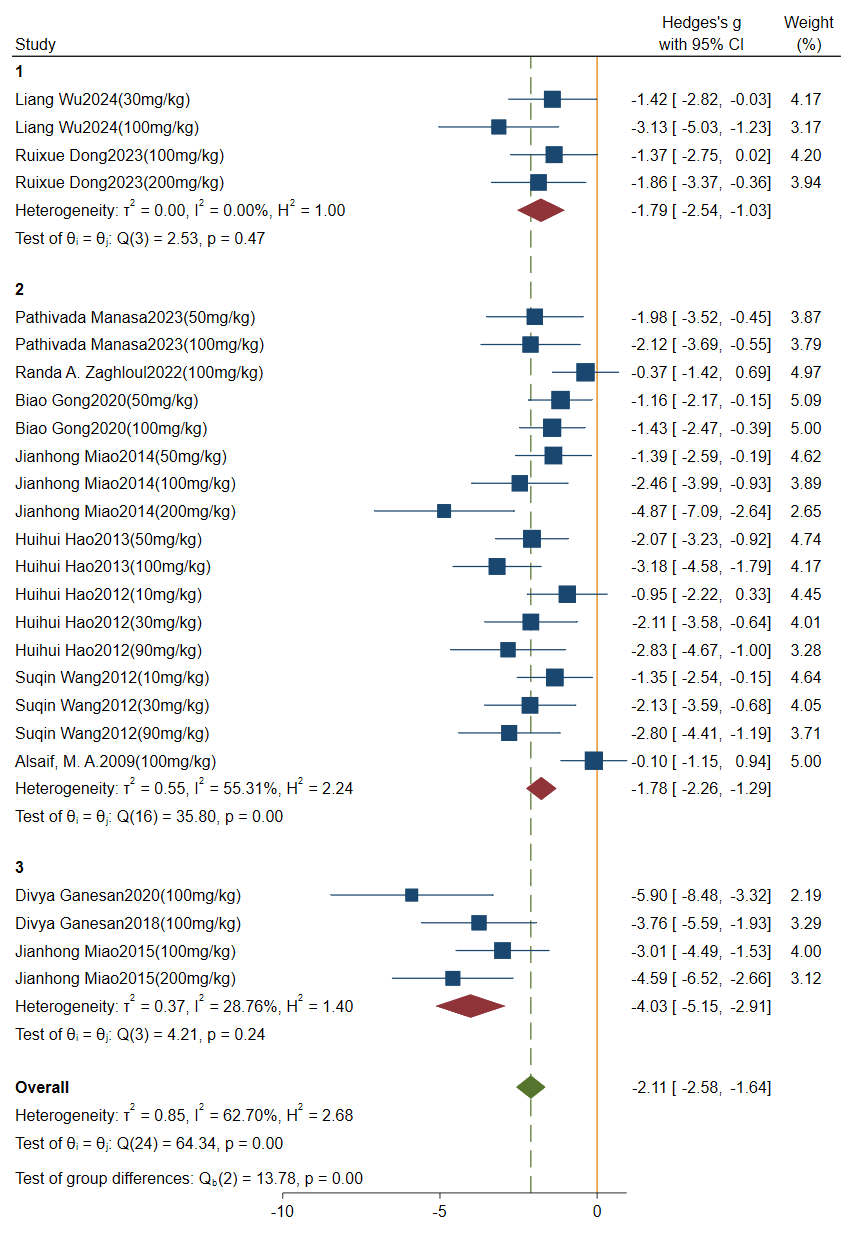
**

**Supplementary Figure 11. Subgroup analysis of Scr based on modeling methodologies**

**
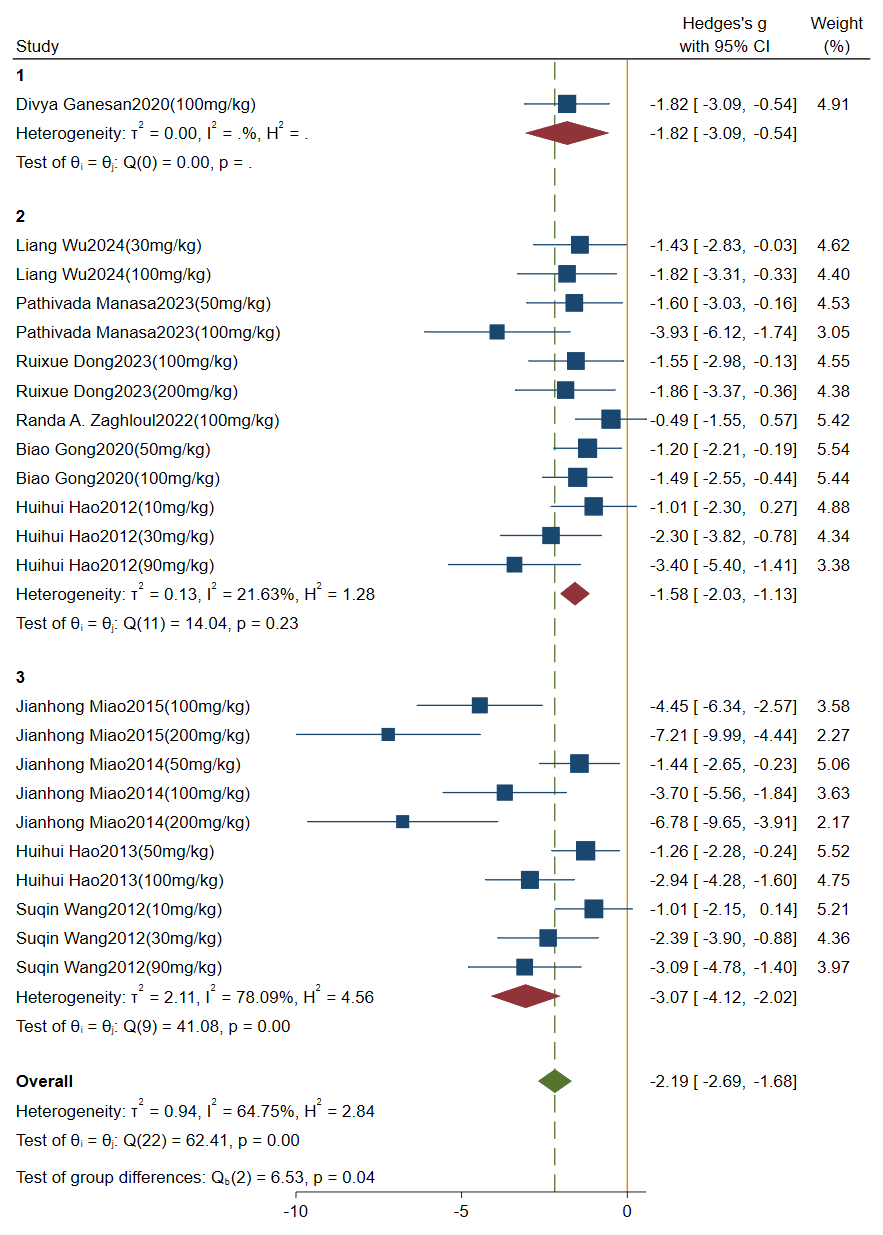
**

**Supplementary Figure 12. Subgroup analysis of BUN based on intervention duration**

**
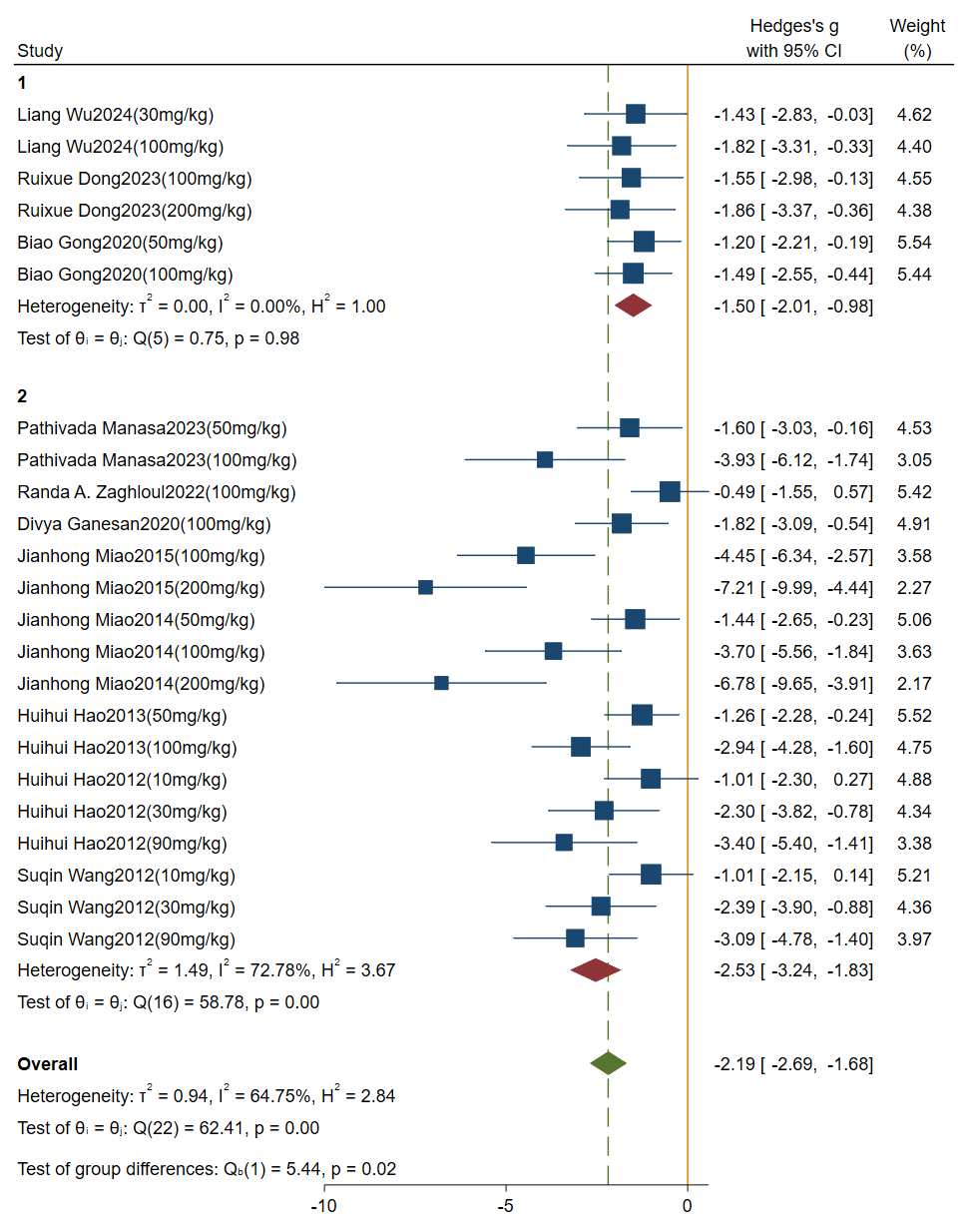
**

**Supplementary Figure 13. Subgroup analysis of BUN based on species of animals**

**
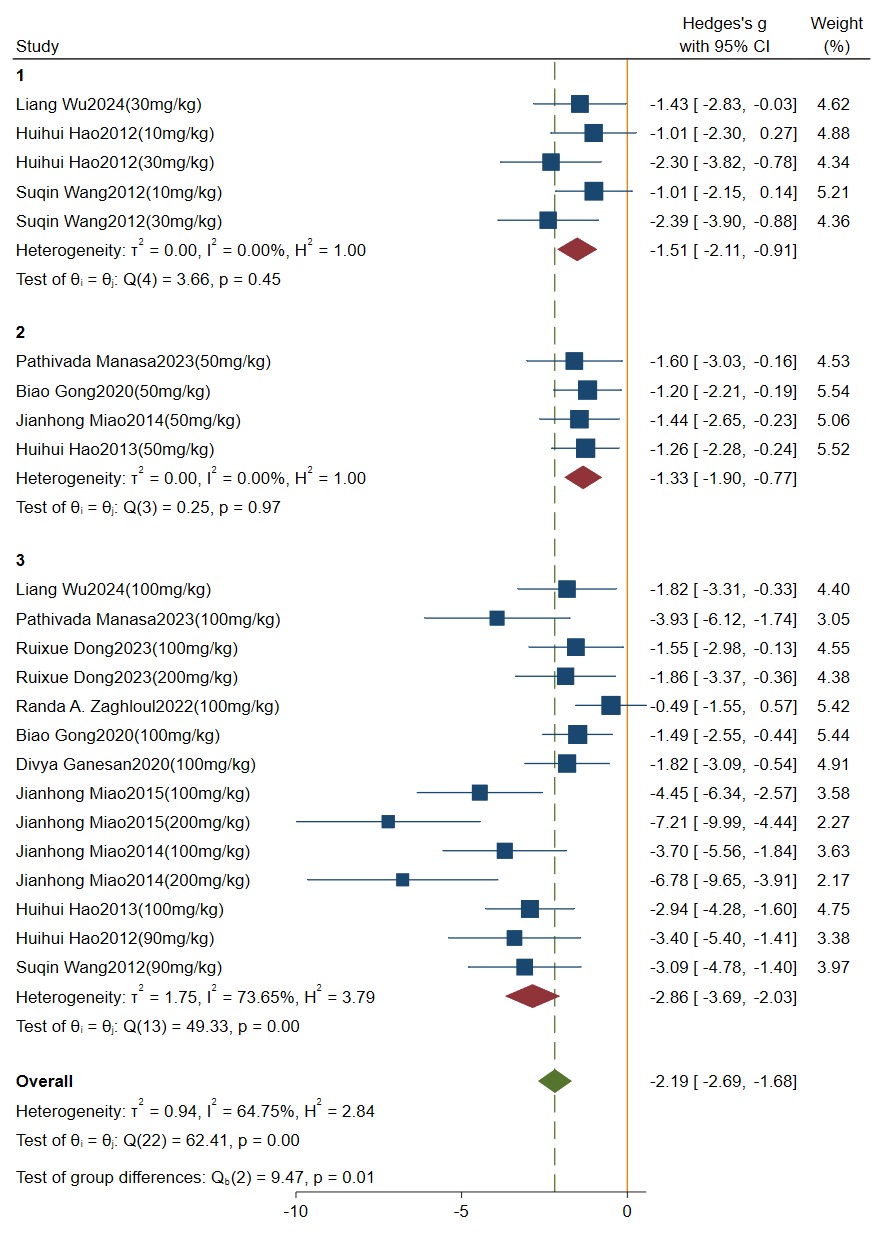
**

**Supplementary Figure 14. Subgroup analysis of BUN based on dosage regimens of rutin**

**
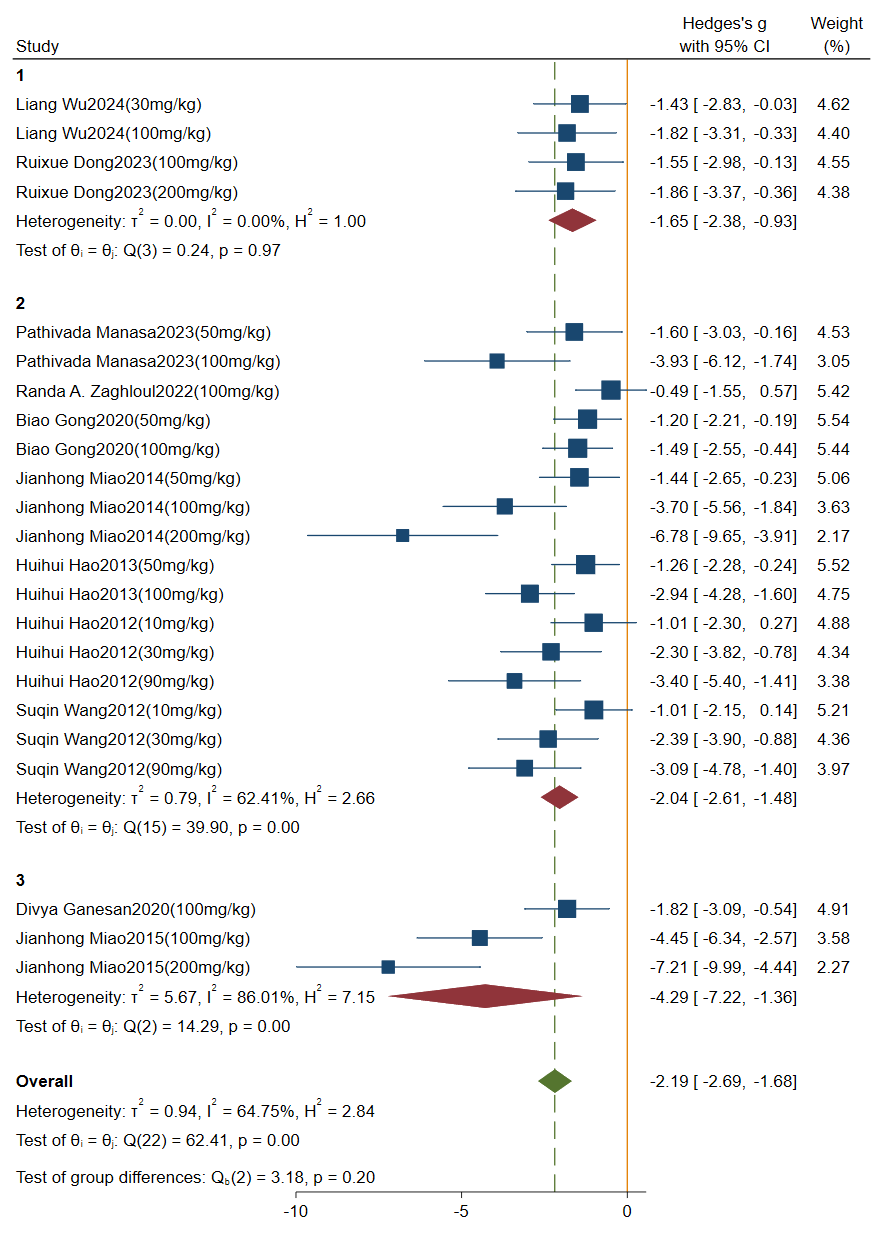
**

**Supplementary Figure 15. Subgroup analysis of BUN based on modeling methodologies**

**
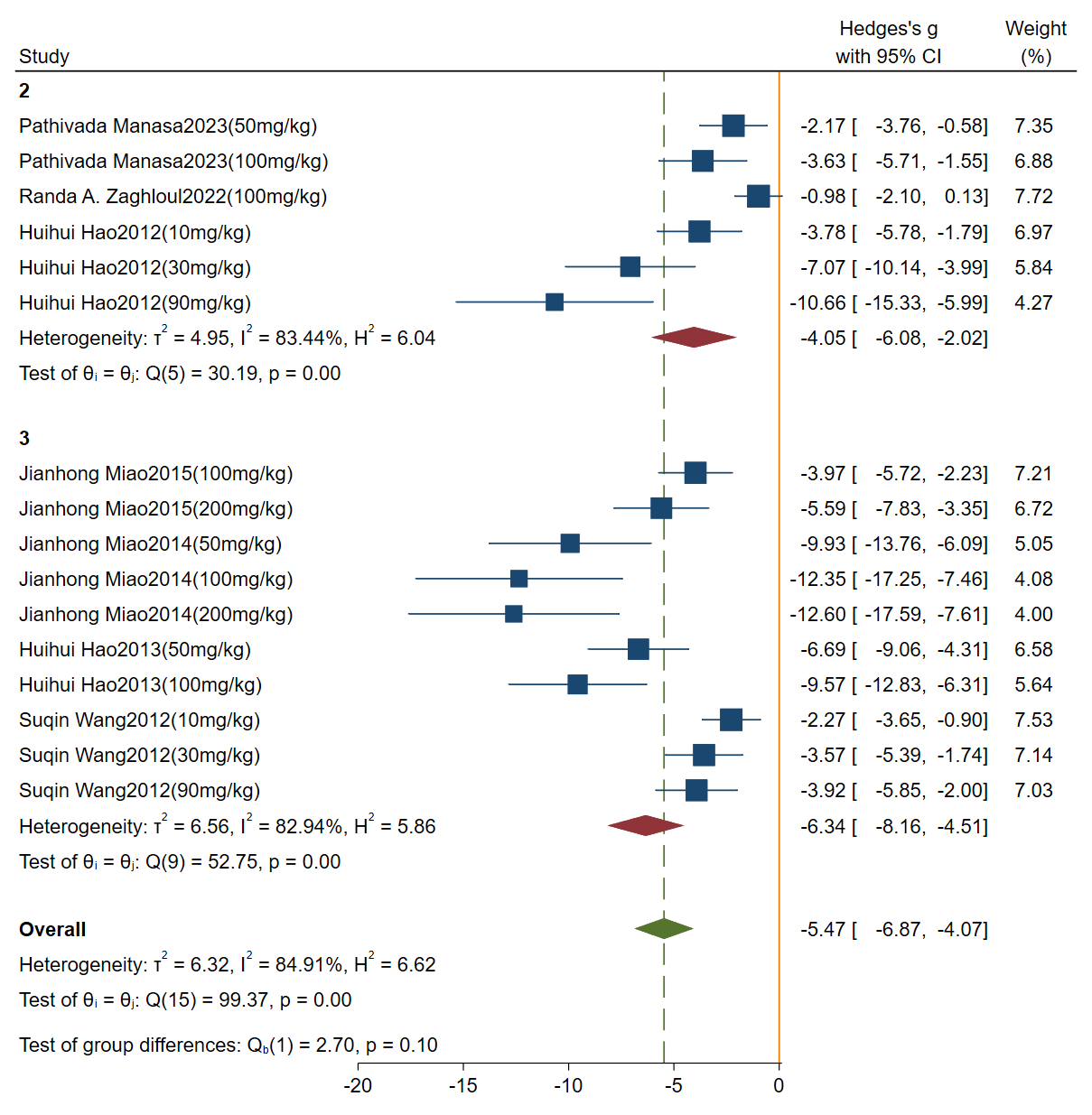
**

**Supplementary Figure 16. Subgroup analysis of 24-h UTP based on intervention duration**

**
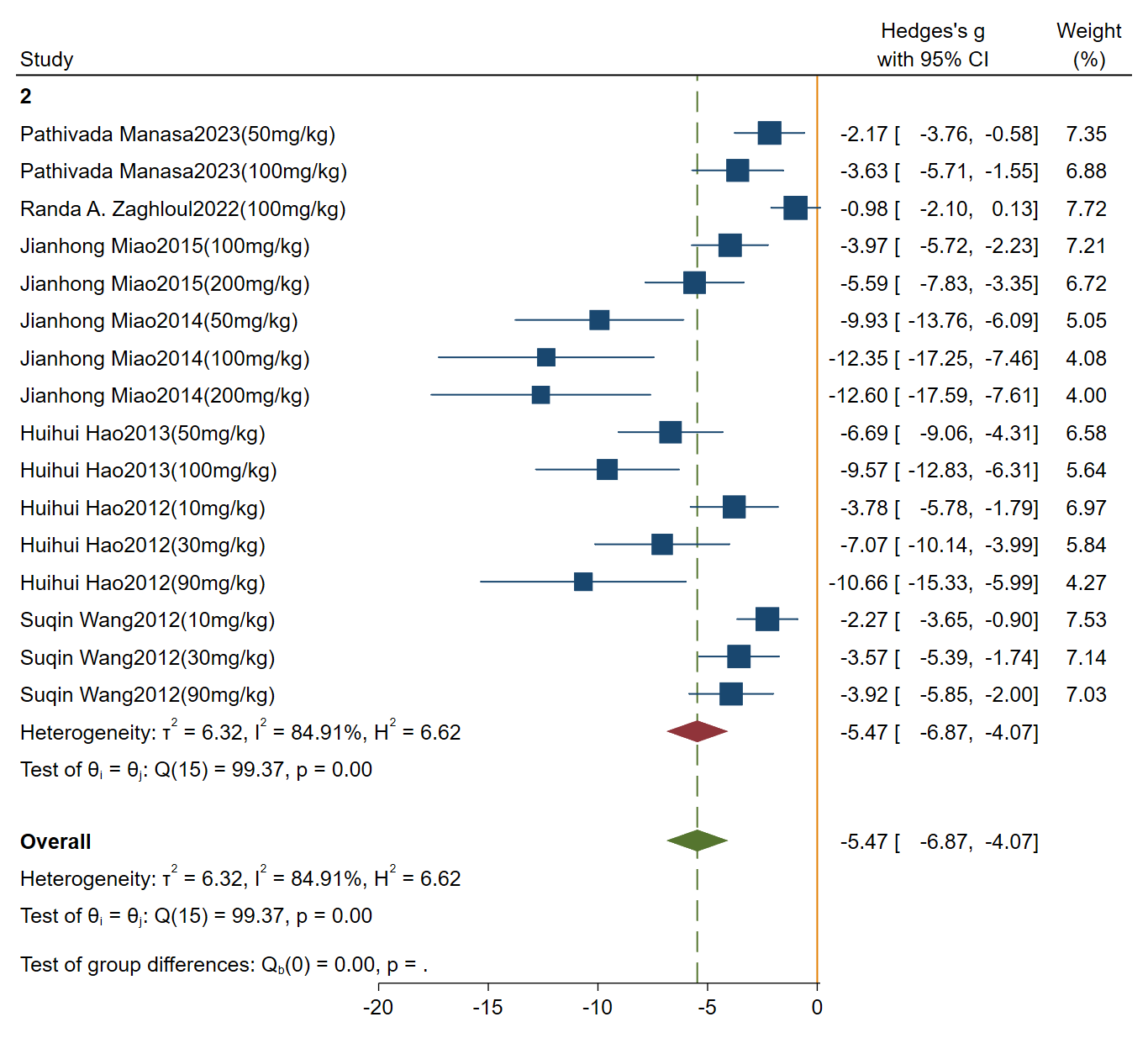
**

**Supplementary Figure 17. Subgroup analysis of 24-h UTP based on species of animals**

**
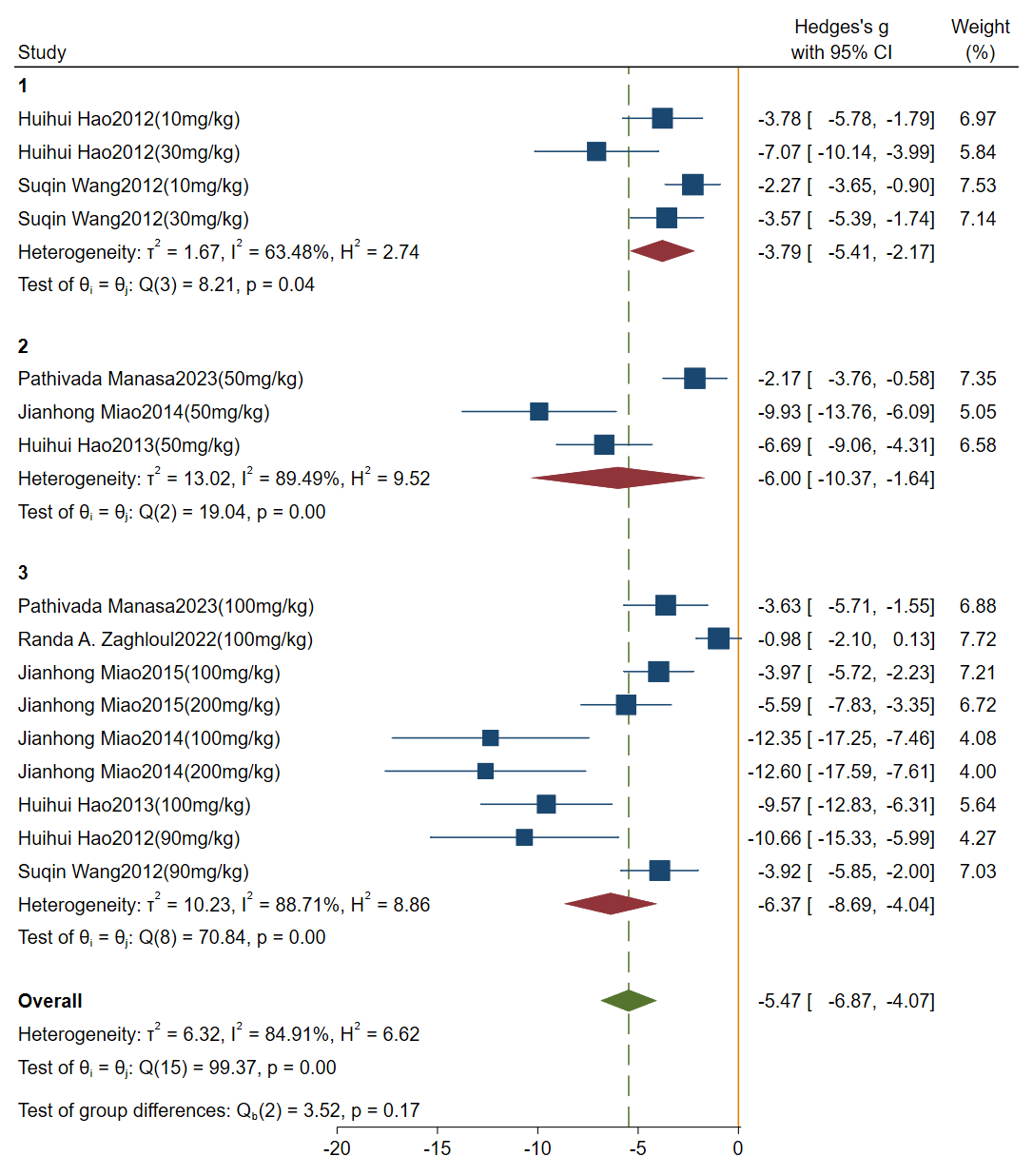
**

**Supplementary Figure 18. Subgroup analysis of 24-h UTP based on dosage regimens of rutin**

**
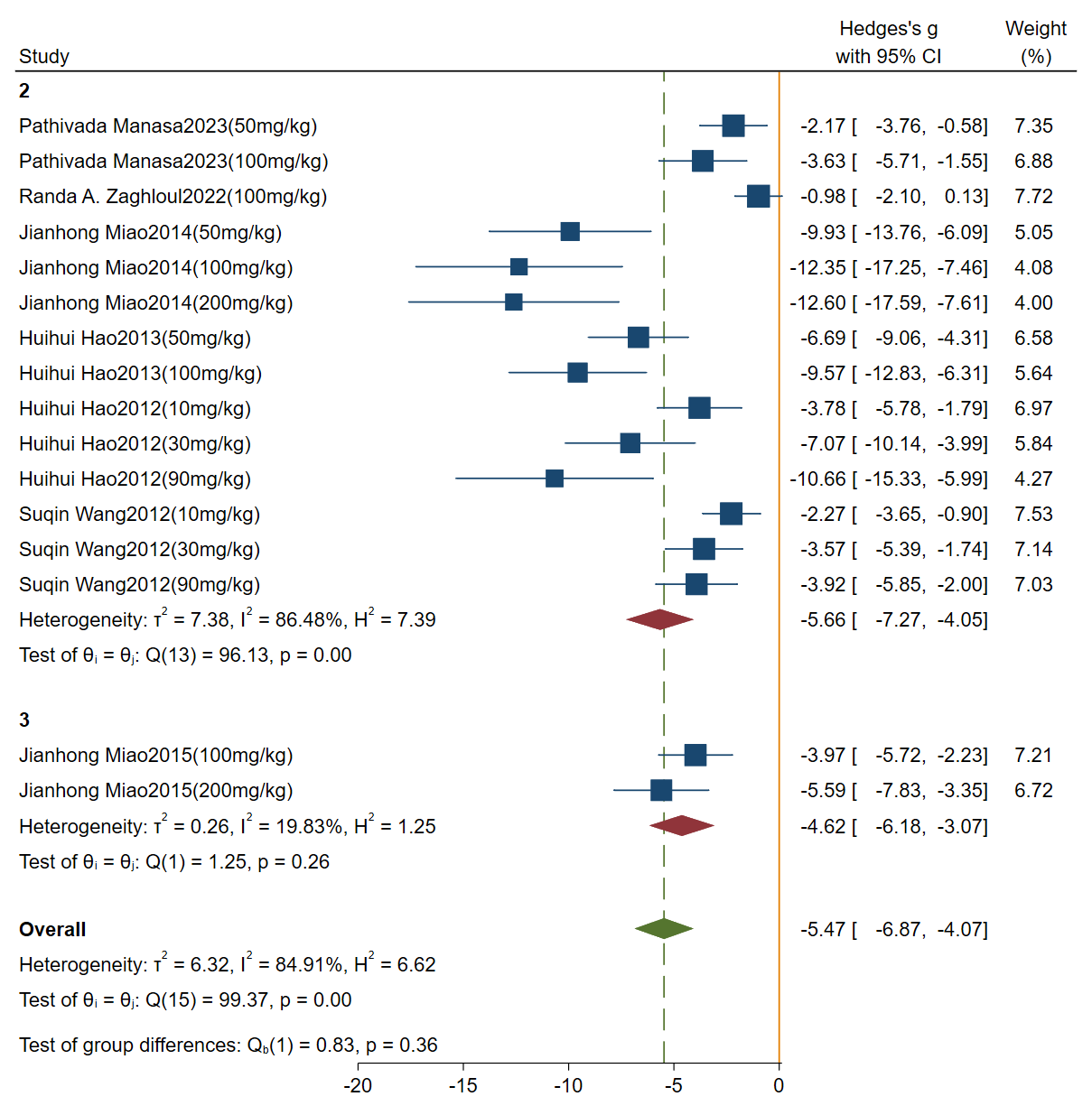
**

**Supplementary Figure 19. Subgroup analysis of 24-h UTP based on modeling methodologies**

**Sensitivity analyses based on studies with directly reported numerical data**

To assess the robustness of the pooled estimates with respect to data source, we first summarized the availability of directly reported numerical data for **primary renal outcomes** across the included studies (Supplementary Table 3). Studies were categorized according to whether outcome data were reported in numerical form (e.g., means and standard deviations in the text or tables) or presented only graphically or not reported. Based on this classification, additional analyses were conducted by restricting inclusion to studies that directly reported outcome data in numerical form. For Scr, BUN, and 24-h UTP, we re-ran outcome-specific meta-analyses using only studies with numerical data (Supplementary Figures 20–22). In addition, leave-one-out sensitivity analyses were performed within these restricted datasets to evaluate the influence of individual studies on the pooled estimates (Supplementary Figures 23–25).

Overall, restricting the analyses to studies with directly reported numerical data yielded pooled effects that were consistent in direction and similar in magnitude to the primary analyses (Supplementary Table **4**). Specifically, the pooled effect for Scr changed from −2.11 (95% CI −2.58 to −1.64) in the primary analysis to −2.38 (95% CI −2.88 to −1.88) in the numerical-data–only analysis; for BUN, from −2.19 (95% CI −2.69 to −1.68) to −2.47 (95% CI −3.08 to −1.85); and for 24-h UTP, from −5.47 (95% CI −6.87 to −4.07) to −5.78 (95% CI −7.15 to −4.40).

These supplementary analyses were conducted to examine whether restricting the data source would materially alter the direction, magnitude, or stability of the estimated effects for the primary renal outcomes.

**Supplementary Table 3. Availability of directly reported numerical data for primary renal outcomes across included studies**

| Study | Scr | BUN | 24-h UTP |
| --- | --- | --- | --- |
| Alsaif, M. A.2009 | × | × | × |
| Huihui Hao2012 | √ | √ | √ |
| Suqin Wang2012 | √ | √ | √ |
| Huihui Hao2013 | √ | √ | √ |
| Jianhong Miao2014 | √ | √ | √ |
| Jianhong Miao2015 | √ | √ | √ |
| Divya Ganesan2018 | √ | × | × |
| Divya Ganesan2020 | √ | √ | × |
| Biao Gong2020 | √ | √ | × |
| Randa A. Zaghloul2022 | × | × | × |
| Ruixue Dong2023 | × | × | × |
| Pathivada Manasa2023 | √ | √ | √ |
| Liang Wu2024 | × | × | × |
| No. of studies (n) | 9 | 8 | 6 |

Note: Studies were categorized according to whether outcome data were directly reported in numerical form (e.g., means and standard deviations in text or tables). A check mark (√) indicates that the corresponding outcome was reported in numerical format and was therefore eligible for inclusion in sensitivity analyses restricted to studies with directly reported data, whereas a cross (×) indicates that the outcome was presented only graphically or not reported.

Supplementary Table 4: Comparison of pooled effects between the primary analysis and the numerical-data–only analysis

| Outcome | Primary analysis  (all eligible studies) (95% CI) | Numerical-data–only analysis  (95% CI) |
| --- | --- | --- |
| Scr | -2.11 [-2.58, -1.64] | -2.38 [-2.88, -1.88] |
| BUN | -2.19 [-2.69, -1.68] | -2.47 [-3.08, -1.85] |
| 24-h UTP | -5.47 [-6.87, -4.07] | -5.78 [-7.15, -4.40] |

**
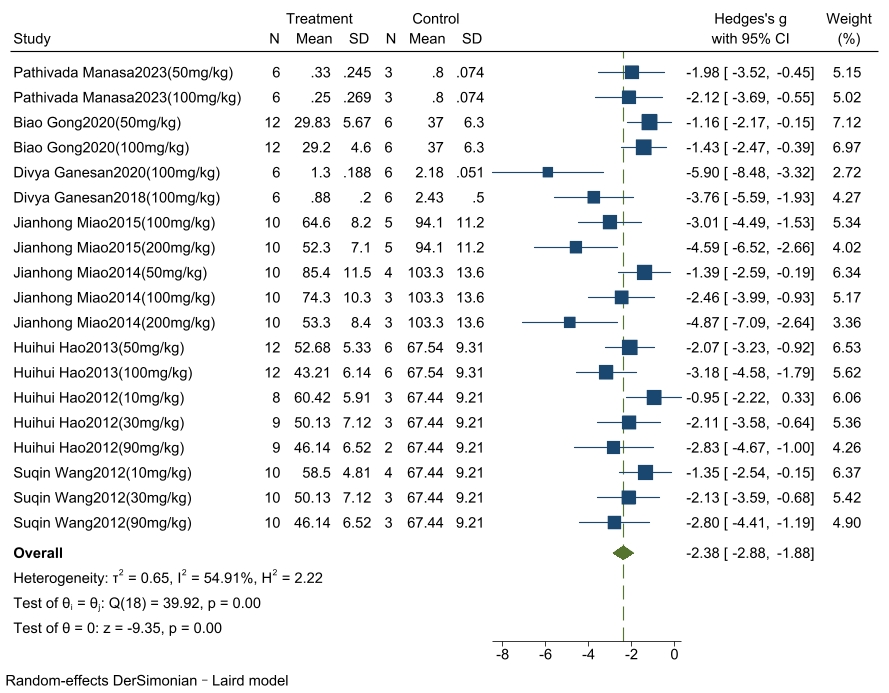
**

**Supplementary Figure 20. Forest plot of pooled effect on Scr restricted to studies with directly reported numerical data.**

**
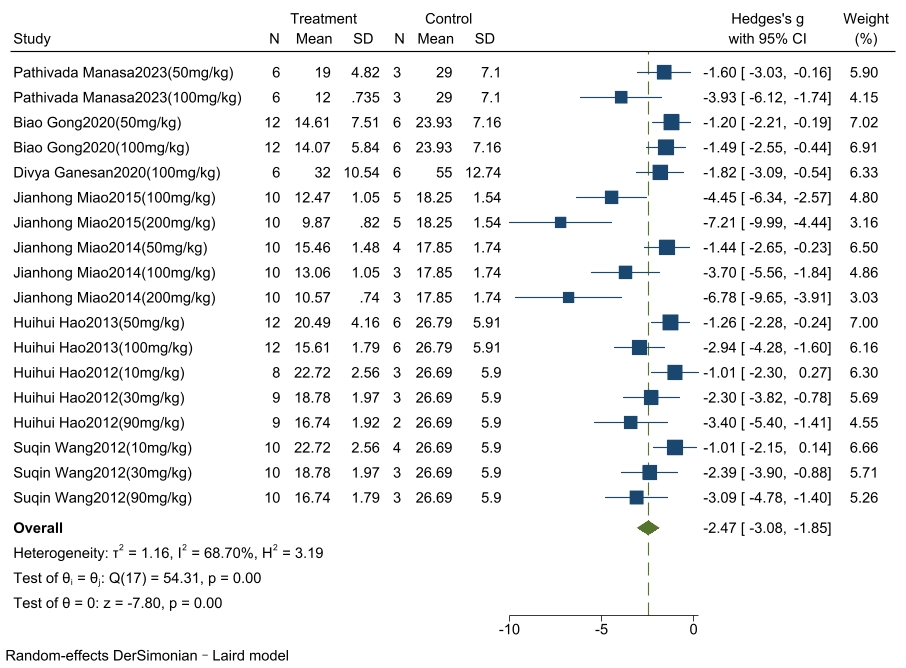
**

**Supplementary Figure 21. Forest plot of pooled effect on BUN restricted to studies with directly reported numerical data.**

**
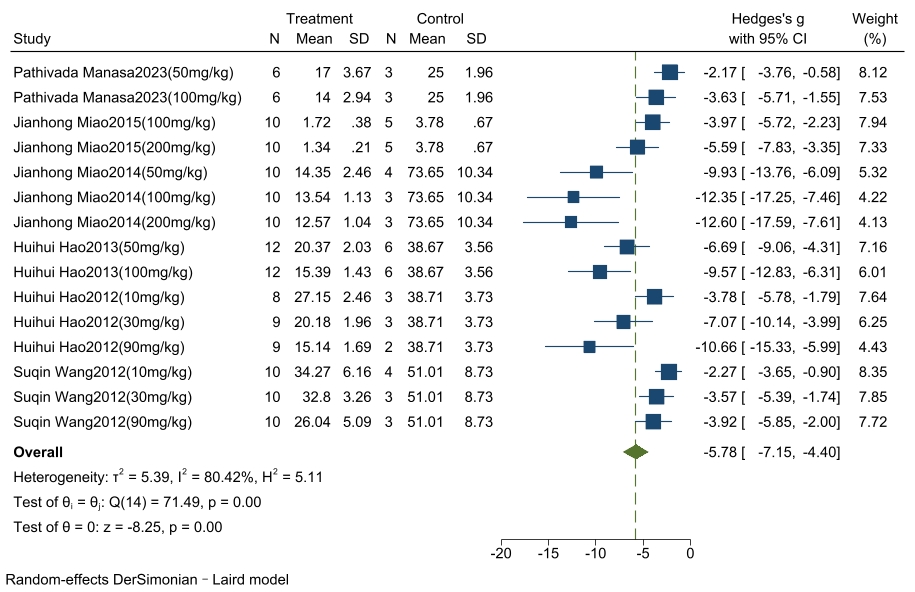
**

**Supplementary Figure 22. Forest plot of pooled effect on 24-h UTP restricted to studies with directly reported numerical data.**


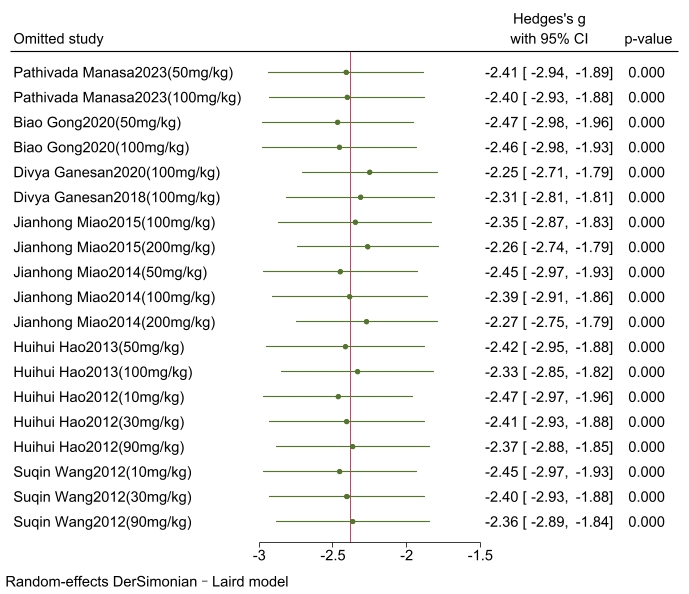


**Supplementary Figure 23**: Leave-one-out sensitivity analysis of Scr restricted to studies with directly reported numerical data


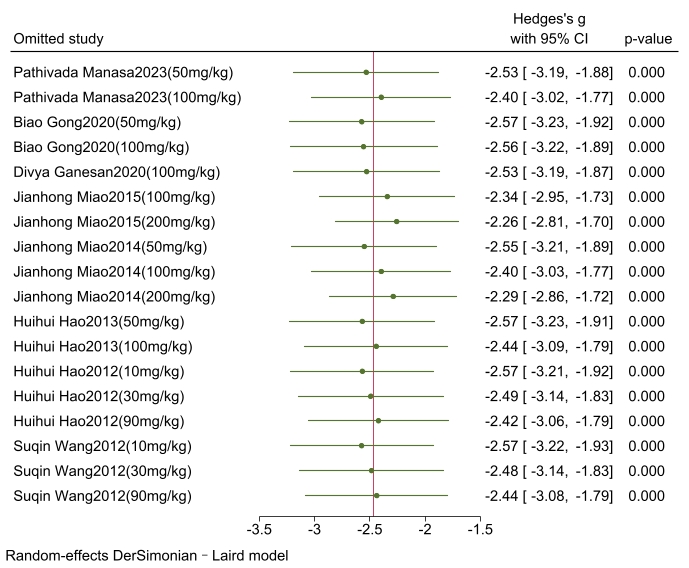


**Supplementary Figure 24**: Leave-one-out sensitivity analysis of BUN restricted to studies with directly reported numerical data


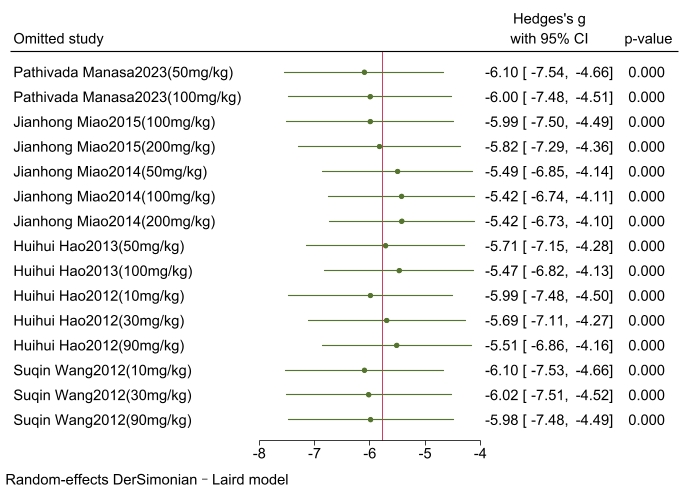


**Supplementary Figure 25**: Leave-one-out sensitivity analysis of 24-h UTP restricted to studies with directly reported numerical data
